# Thienopyrimidine: A Promising Scaffold to Access Anti-Infective Agents

**DOI:** 10.3390/ph15010035

**Published:** 2021-12-27

**Authors:** Prisca Lagardère, Cyril Fersing, Nicolas Masurier, Vincent Lisowski

**Affiliations:** 1 IBMM, University of Montpellier, CNRS, ENSCM, 34293 Montpellier, France; prisca.lagardere@umontpellier.fr (P.L.); cyril.fersing@umontpellier.fr (C.F.); vincent.lisowski@umontpellier.fr (V.L.); 2 Nuclear Medicine Department, Montpellier Cancer Institute (ICM), University of Montpellier, 208 Avenue des Apothicaires, CEDEX 5, 34298 Montpellier, France; 3 Department of Pharmacy, Lapeyronie Hospital, CHU Montpellier, 191 Av. du Doyen Gaston Giraud, 34295 Montpellier, France

**Keywords:** thienopyrimidine, antibacterial, antifungal, antiparasitic, antiviral

## Abstract

Thienopyrimidines are widely represented in the literature, mainly due to their structural relationship with purine base such as adenine and guanine. This current review presents three isomers—thieno[2,3-*d*]pyrimidines, thieno[3,2-*d*]pyrimidines and thieno[3,4-*d*]pyrimidines—and their anti-infective properties. Broad-spectrum thienopyrimidines with biological properties such as antibacterial, antifungal, antiparasitic and antiviral inspired us to analyze and compile their structure–activity relationship (SAR) and classify their synthetic pathways. This review explains the main access route to synthesize thienopyrimidines from thiophene derivatives or from pyrimidine analogs. In addition, SAR study and promising anti-infective activity of these scaffolds are summarized in figures and explanatory diagrams. Ligand–receptor interactions were modeled when the biological target was identified and the crystal structure was solved.

## 1. Introduction

**Figure 1:**
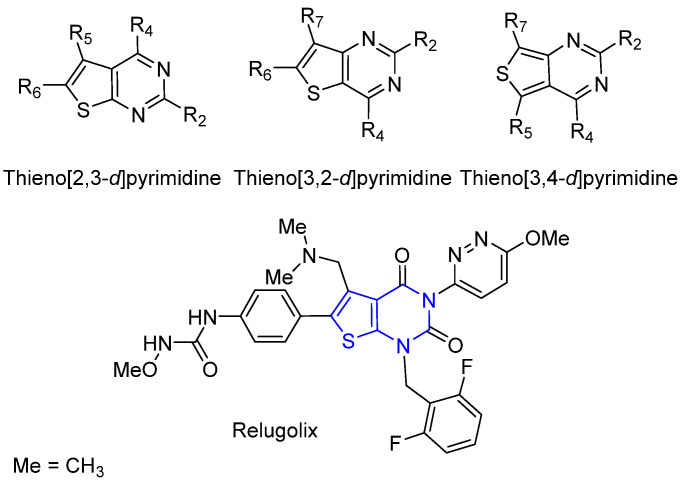
Structure of thienopyrimidines and of Relugolix (the thienopyrimidine scaffold is highlighted in blue).

In recent years, thieno-fused derivatives are of growing interest and are found in many original bioactive molecules [1,2], even if the thiophene ring is known to potentially generate reactive metabolites [3]. Among thieno-fused derivatives, thienopyrimidines have been widely studied in the literature, probably due to their structural relationship with purine bases and their easy synthetic access. Considering the fusion between pyrimidine and thiophene rings, three different thienopyrimidines can be obtained, namely thieno[2,3-*d*]pyrimidines, thieno[3,2-*d*]pyrimidines and thieno[3,4-*d*]pyrimidines (Figure 1). All three have been studied and many derivatives have shown a large range of biological activities, such as anticancer, antioxidant, and central nervous system (CNS) protection. Some of them are still in clinical trials [2], while others have even reached the market (e.g., Relugolix, a gonadotropin-releasing hormone (GnRH) receptor antagonist, Figure 1).

In a recent review, Ali et al. summed up the biological activities of the thieno[2,3-*d*]pyrimidine scaffold until the end of 2018, with a particular attention provided onto their anticancer activities [2]. Due to our interest in the development of new anti-infective compounds [4,5,6,7,8,9], the objective of the present review is to provide an overview of the access routes to thienopyrimidine derivatives and to discuss the significance of this scaffold for the discovery of anti-infective drugs. In this review, we have collected all references until September 2021 involving the three isomers presented above with anti-infective properties. Only compounds with quite similar or higher activities compared to the selected reference drugs are presented.

## 2. Synthesis of Thienopyrimidines

Different synthetic pathways involving the construction of the pyrimidine or the thiophene ring were reported in the literature to access polysubstituted thienopyrimidines. In these approaches, the synthetic strategies mostly involved the synthesis of a thienopyrimidin-4-one derivative, where position 4 could be modified via further functionalization.

### 2.1. Synthesis from Thiophene Derivatives

**Scheme 1:**
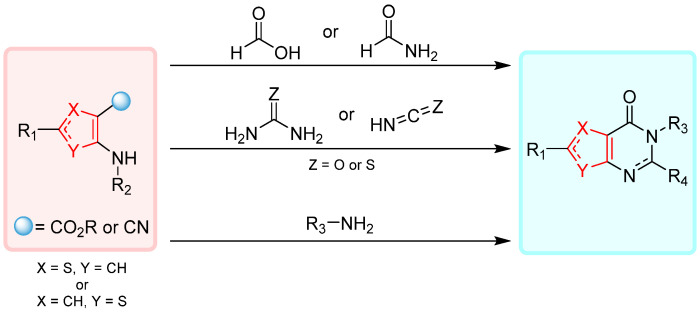
Main synthetic pathways to produce thienopyrimidin-4-ones from thiophene derivatives.

Due to the high diversity of supplies, the reaction between an aminothiophene derivative bearing an electrophilic center (ester or nitrile) and a carbonyl or an amine reactant is probably the easiest way for produce thienopyrimidin-4-one derivatives. The leading routes to afford thienopyrimidines from aminothiophene derivatives are described in Scheme 1.

#### 2.1.1. Cyclization with Carbonyl Reactants

**Scheme 2:**
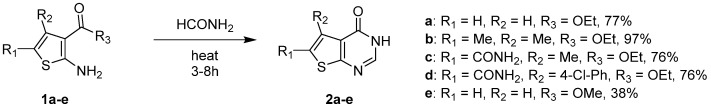
Access to 2- and 3-unsubstituted thieno[2,3-*d*]pyrimidin-4-one derivatives (Me = methyl, Et = ethyl, and Ph = phenyl).

The most efficient chemical approach to access 2- and 3-unsubstituted thieno[2,3-*d*]pyrimidin-4(3*H*)-ones involved a condensation reaction between an aminothiophene substrate and formamide. Thus, compounds **1a–e** treated with an excess of formamide at high temperature led to compounds **2a–e** with good yields (76 to 97%), except for compound **1e** for which the methoxy group in R_3_ decreased the reaction yields compared to the ethoxy group (**1a**) (Scheme 2) [10,11,12,13,14].

**Scheme 3:**
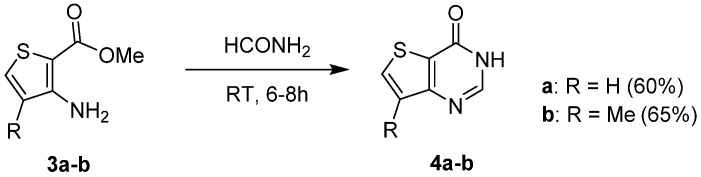
Synthesis of 3-unsubstituted thieno[3,2-*d*]pyrimidines **4a–b**.

In contrast, mild conditions were sufficient to perform cyclization reaction with formamide to synthesize the thieno[3,2-*d*]pyrimidin-4(3*H*)-one isomers **4a–b** with good yields (60 to 65%, Scheme 3) [15].

**Scheme 4:**
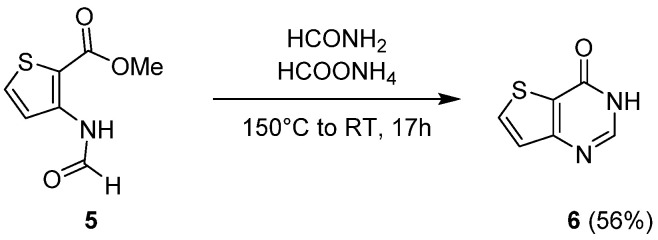
Synthetic route to unsubstituted thieno[3,2-*d*]pyrimidin-4-one **6**.

Woodring et al. presented a variant of this process that also involved formamide in combination with ammonium formate [14]. Cyclization of the thiophene intermediate **5** at 150 °C led to the unsubstituted thieno[3,2-*d*]pyrimidin-4-one **6** with a 56% yield (Scheme 4).

**Scheme 5:**
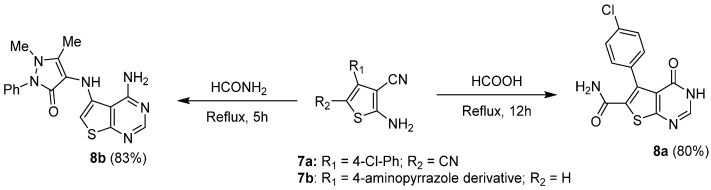
Access to thieno[2,3-*d*]pyrimidine derivatives from 2-amino-3-cyanothiophene derivatives.

In addition, reaction of 2-amino-3-cyanothiophene derivatives with formic acid could also be considered to access 2- and 3-unsubstituted thieno[2,3-*d*]pyrimidin-4-ones [13]. In such approach, the cyano group is firstly converted into its corresponding primary amide, which could then be cyclized in the presence of formic acid. Kanawade et al. used such an approach to prepare thienopyrimidinone **8a** from 2-amino-3,5-dicyanothiophene **7a** (Scheme 5). Replacing formic acid by formamide led to the formation of the 4-amino analogue, as reported by Aly et al. [16]. Thus, cyclocondensation involving **7b** and formamide occurred under reflux to afford the expected **8b** with a 83% yield (Scheme 5).

**Scheme 6:**
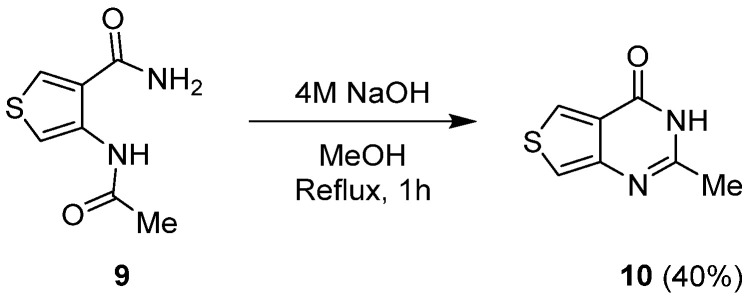
Synthesis of 2-methyl-thieno[3,4-*d*]pyrimidin-4(3*H*)-one **10**.

Cyclocondensation of thiophene carboxamide **9** in the presence of sodium hydroxide was used to synthesize thieno[3,4-*d*]pyrimidin-4(3*H*)-one **10** (Scheme 6). The expected molecule was isolated with a moderate yield (40%) after a 1 h reaction in refluxing methanol.

**Scheme 7:**
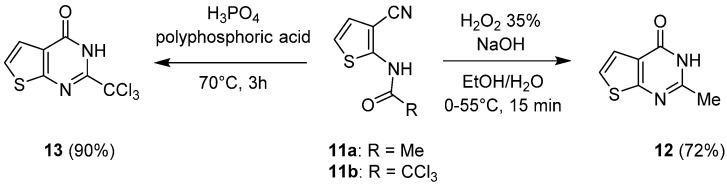
Synthesis of thieno[2,3-*d*]pyrimidin-4(3*H*)-ones substituted in position 2.

Using a similar approach, but with a nitrile group as the precursor of the primary amide, Desroches et al. synthesized 2-methyl- and 2-trichloromethyl-thieno[2,3-*d*]pyrimidin-4(3*H*)-ones **12** and **13**, respectively (Scheme 7) [17]. Thus, treatment of 3-cyanothiophene acetamide **11a** with hydrogen peroxide in alkaline medium (NaOH) afforded 2-methyl-thieno[2,3-*d*]pyrimidin-4(3*H*)-one **12** with a 72% yield. Using 3-cyanothiophene trichloroacetamide as a substrate and phosphoric acid in polyphosphoric acid triggered the cyclocondensation reaction and the formation of the 2-trichloromethyl-thieno[2,3-*d*]pyrimidin-4(3*H*)-one **13** with good yields (90%).

#### 2.1.2. Cyclization with Nitrile Reactants

**Scheme 8:**
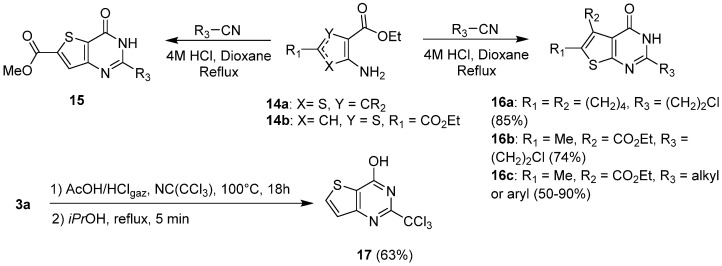
Synthesis of 2-substituted thienopyrimidin-4-ones using nitrile reactants.

Various pathways exploiting nitrile condensation were reported in the literature to produce thieno-fused analogues. De Schutter et al. used a synthetic route involving a thiophene amino ester treated in strongly acidic conditions by a cyanoalkyl derivative at 90 °C (Scheme 8) [18]. Thieno[2,3-*d*]pyrimidin-4(3*H*)-ones **16c**, substituted in positions 2, 5, and 6 were then obtained in 1,4-dioxane in moderate to good yields (50 to 90%). In addition, Mavrora et al. used the same synthetic pathway and obtained chloroethyl derivatives **16a–b** with good yields (Scheme 8) after nitrile cyclocondensation at room temperature [19]. Likewise, thieno[3,2-*d*]pyrimidinones **15** substituted at position 2 were prepared from cyclization of the starting thiophene with the appropriate cyanoalkyl in acidic conditions at 90 °C in 1,4-dioxane (Scheme 8) [18]. To introduce a trichloromethyl group at position 2 of the thieno[3,2-*d*]pyrimidine core, Desroches et al. used trichloroacetonitrile in acetic acid, saturated with HCl gas, to afford 2-trichloromethyl-thieno[3,2-*d*]pyrimidine **17** with a 63% yield (Scheme 8) [17].

**Scheme 9:**
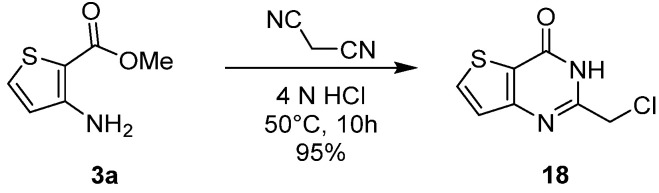
Synthesis of 2-chloromethyl-thieno[3,2-*d*]pyrimidinone **18**.

Using the same strategy, Kim et al. introduced a chloromethyl group at position 2 of thieno[3,2-*d*]pyrimidinones after slight modifications of the reaction conditions [20]. Formation of the thieno-fused core occurred with the cyclocondensation of malononitrile with 2-methyl-3-aminothiophene carboxylate under acidic conditions and mild heating to offer **18** with high yields (Scheme 9).

**Scheme 10:**
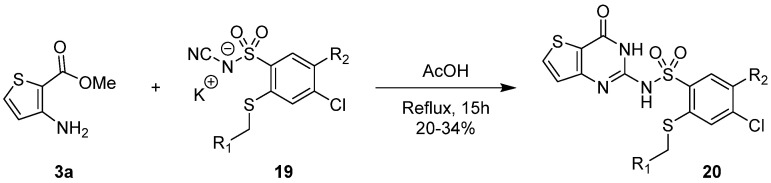
Formation of 2-sulfonamide-thieno[3,2-*d*]pyrimidinones **20**.

Slavinski et al. presented another synthetic pathway to introduce a sulfonamide group at position 2, using sulfonyl cyanamide potassium salts **19** [21]. Acidification of the reaction with boiling glacial acetic acid led to cyclization and afforded 2-sulfonamide-thieno[3,2-*d*]pyrimidinone derivatives **20** with low yields (20–34%, Scheme 10).

#### 2.1.3. Synthesis from (Thio)urea Reagents, Iso(Thio)cyanate or (Thio)cyanate Derivatives

**Scheme 11:**
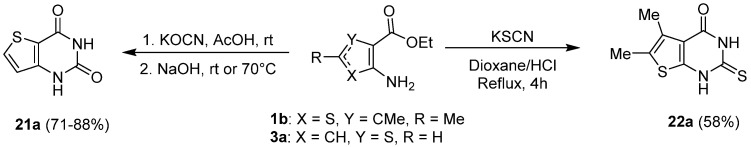
Synthesis of 2-thioxo-thieno[2,3-*d*]pyrimidin-4-one **22a** and thieno[3,2-*d*]pyrimidin-2,4-dione **21a**.

An easy way to access thienopyrimidin-2,4-dione or 2-thioxo-thienopyrimidin-4-one derivatives consisted of cyclocondensation of the appropriate ethyl aminothiophene-carboxylate with potassium (thio)cyanate in an acidic medium. Patel et al. obtained 2-thioxo-thieno[2,3-*d*]pyrimidin-4-one **22a** with a 58% yield, using hydrochloric acid in refluxing 1,4-dioxane (Scheme 11) [22], whereas Temburkinar et al. and other groups [23,24,25] used potassium cyanate in acetic acid to obtain thieno[3,2-*d*]pyrimidin-2,4-dione **21a** with 71 to a 88% yield.

**Scheme 12:**
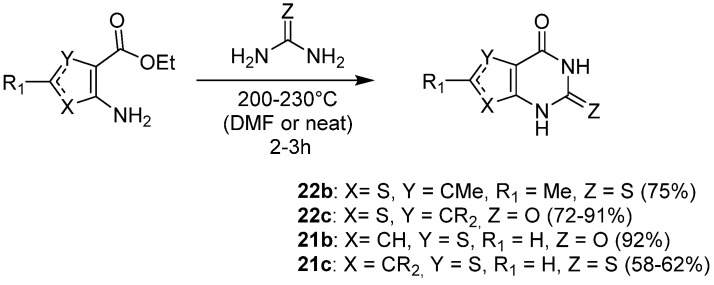
Formation of 2-thioxo-thienopyrimidin-4-ones and thienopyrimidine-2,4-diones using (thio)urea.

Another way to access such compounds was to condensate the starting aminothiophene with urea or thiourea, followed by cyclization to afford thienopyrimidinone compounds **21** or **22**. Ortikov and Prabhakar teams used such conditions to synthesize 2-thioxo-thieno[2,3-*d*]pyrimidin-4-one **22b** and thieno[2,3-*d*]pyrimidine-2,4-diones **22c** (Scheme 12) with good yields (72–91%) [11,26,27]. Condensation and cyclization only occurred at very high temperatures after 2 or 3 h of heating without solvent. Thieno[3,2-*d*]pyrimidin-2,4-one **21b** could be synthesized under these conditions, whereas the synthesis of 2-thioxo-thieno[3,2-*d*]pyrimidin-4-ones **21c** required the use of *N*,*N*-dimethylformamide (DMF) as a solvent (Scheme 12) [28,29].

**Scheme 13:**
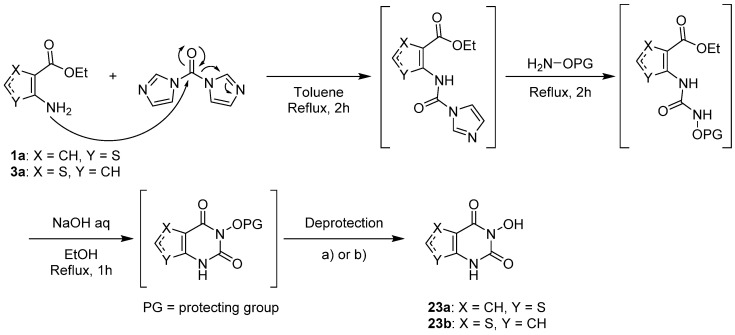
Synthetic pathway to afford 3-hydroxythienopyrimidin-2,4-diones **23**.

Kankanala et al. used a common synthetic pathway to access 3-hydroxythieno[2,3-*d*]pyrimidin-2,4-diones and thieno[3,2-*d*]pyrimidin-2,4-diones [30] bearing various groups in α and β positions of the sulfur atom. Firstly, the aminothiophene reacted with 1,1′-carbonyldiimidazole (CDI) to afford the imidazole-carboxamide intermediate after 2 h in refluxing toluene (Scheme 13). Secondly, the substitution of the imidazole group by protected hydroxylamine generated the hydroxyurea intermediate. Then, a basic treatment deprotonated hydroxyurea to allow cyclization. Afterward, deprotection of the hydroxyurea led to the final compounds **23** with correct to good yields (40–85%).

**Scheme 14:**
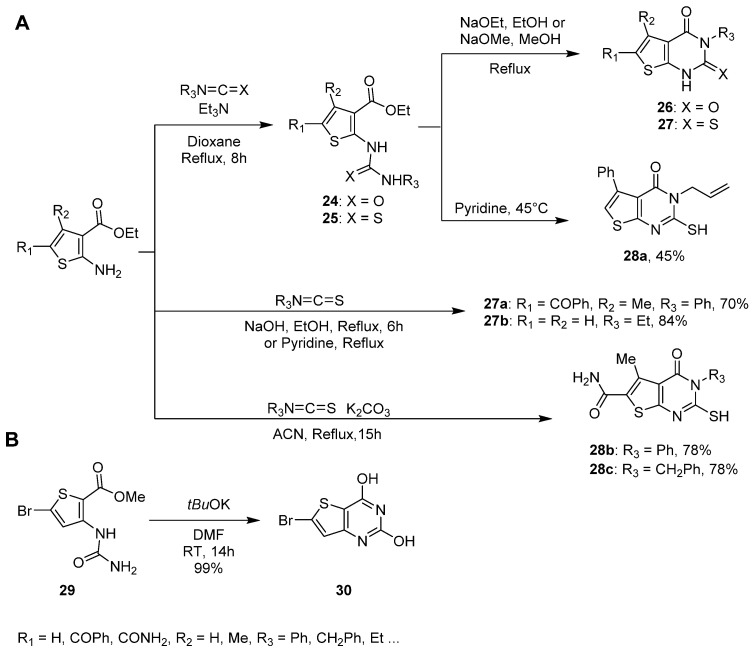
(**A**). Synthesis of 3-substituted 2-thioxo-thienopyrimidin-4-ones or thienopyrimidine-2,4-diones **26**–**28**. (**B**). Synthesis of 6-bromothieno[3,2-*d*]pyrimidine-2,4-diol **30**.

To introduce more chemical diversity at position 3, a convenient synthetic route described by Abu-Hashem et al. involved nucleophilic attack of an aminothiophene derivative on an isocyanate or thioisocyanate in the presence of a catalytic amount of triethylamine in refluxing 1,4-dioxane (Scheme 14A) [31]. The (thio)ureidothiophene intermediate **24** or **25** was then isolated on average with good yields (60 and 70%). Thereafter, basic treatment of **24** or **25** with sodium ethoxide in refluxing ethanol led to thieno-fused derivatives **26** and **27** with good yields (70% and 75%) after 8 h. Dewal et al. obtained similar results using sodium methoxide under refluxing methanol to prepare trisubstituted thieno[2,3-*d*]pyrimidin-2,4-dione derivatives **26** with 88–90% yields [32]. In addition, Abu-Hashem et al. reported a one-pot reaction with phenylisothiocyanate and sodium hydroxide as a base, in refluxing ethanol for 6 h [31]. Both the two-step procedure and the one-pot reaction offered **27a** with a 70% yield (Scheme 14A). Furthermore, the use of potassium carbonate in refluxing acetonitrile led to the 2-mercapto-thieno[2,3-*d*]pyrimidin-4-one analogues **28b–c** in even higher yields (78%) [12,33]. In a similar way, 3-ethyl-2-thioxo-thieno[3,2-*d*]pyrimidin-4-one **27b** was also accessible via the cyclization of 2-methyl-3-aminothiophene carboxylate with ethylisothiocyanate in refluxing pyridine [34]. In addition, 6-bromothieno[3,2-*d*]pyrimidin-2,4-diol **30** was synthesized in milder conditions with potassium *tert*-butoxide in DMF at room temperature and obtained it with a quantitative yield (Scheme 14B) [35]. It was then possible to introduce further chemical diversity in positions 2, 4, and 6, starting from this bicyclic product.

**Scheme 15:**
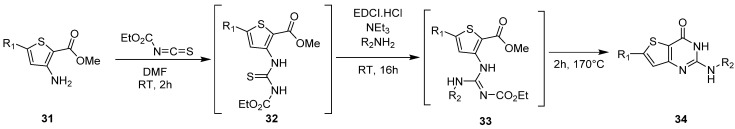
Synthetic pathway purposed by Cohen et al. [36].

Alternately, Cohen et al. suggested an original synthetic pathway to obtain thieno[3,2-*d*]pyrimidin-4(3*H*)-one derivatives **34**, substituted in position 2 by an amino group [36]. This one-pot procedure involved first the condensation of the starting material with ethoxycarbonyl isothiocyanate in DMF to generate the thiourea carbamate intermediate **32**, that was not isolated (Scheme 15). Afterward, a primary alkylamine reacted with this species, previously mixed with 1-ethyl-3-(3′-dimethylaminopropyl)carbodiimide (EDCI.HCl) and triethylamine. Guanidine intermediate **33** was observed but was not isolated. Then, this intermediate cyclized at 170 °C to afford thieno-fused derivatives **34** with 42 to 70% yields depending on the substituents.

#### 2.1.4. Synthesis via a Tetrazole Intermediate

**Scheme 16:**
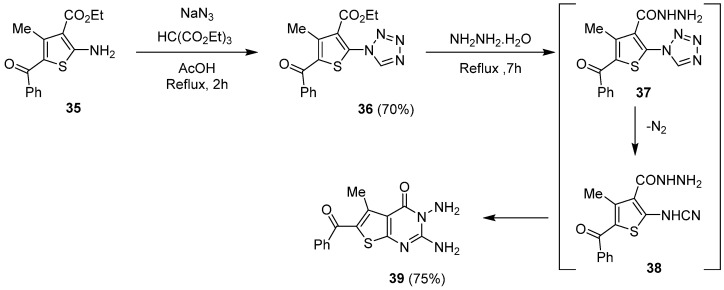
Synthesis of 2,3-diaminothieno[2,3-*d*]pyrimidine **39**.

To generate thieno[2,3-*d*]pyrimidines substituted in positions 2 and 3 by an amino group, Abu-Hashem et al. purposed an access route via a tetrazole intermediate (Scheme 16) [31]. Firstly, the tetrazole ring was formed by treating **35** with triethyl orthoformate and sodium azide to generate **36** with good yields (70%). Then, refluxing **36** in the presence of a large excess of hydrazine hydrate led to two consecutive hydrazide intermediates **37** and **38**. Intramolecular cyclization of **38** afforded **39** with good yields (75%).

#### 2.1.5. Cyclization with Amine/Hydrazine Derivatives

**Scheme 17:**
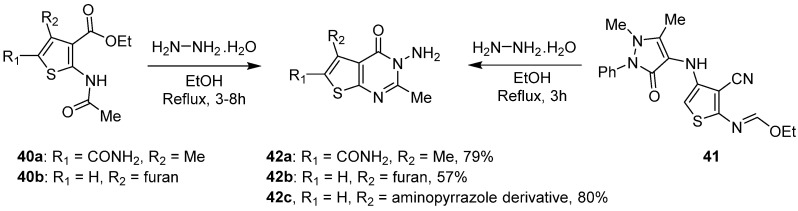
Synthesis of 3-amino-thienopyrimidin-4-ones **42**.

A more common way to access 3-amino-thieno[2,3-*d*]pyrimidin-4-ones consisted of the condensation and cyclization between a thiophene derivative and hydrazine monohydrate in refluxing ethanol. Using this strategy, several groups reported the synthesis of compounds **42a–b** with moderate to good yields (Scheme 17) [12,37]. Aly et al. employed the same reaction conditions to generate 3-amino-thieno[2,3-*d*]pyrimidin-4-one **42c**. Only the starting thiophene was different and achieved cyclocondensation with good yields (80%).

**Scheme 18:**
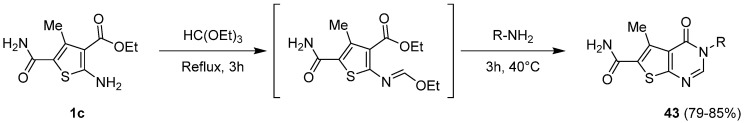
Access route to synthesize 3-substituted thieno[2,3-*d*]pyrimidin-4-ones **43**.

To introduce chemical diversity at position 3, a similar route was followed by Habib et al. using various primary amines to synthesize a set of 3-substituted thieno[2,3-*d*]pyrimidinone derivatives **43** [12]. Firstly, the 2-aminothiophene **1c** reacted with triethyl orthoformate under reflux to prepare the imino intermediate, which was not isolated (Scheme 18). Then, the appropriate amine was added to allow cyclization and obtain 3-substituted thienopyrimidinone derivatives **43** with good yields (79–85%).

**Scheme 19:**
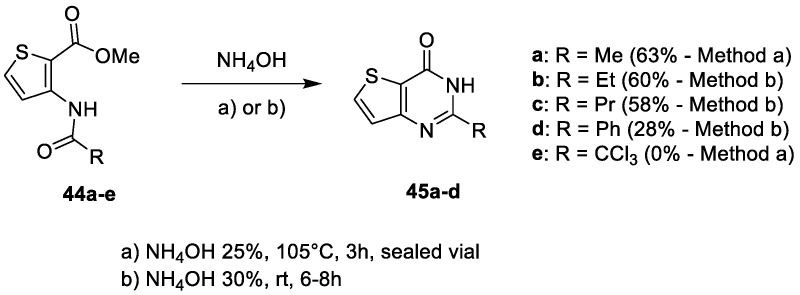
Synthesis of 3-unsubstituted-thienopyrimidin-4-ones **45** (Pr = propyl).

Finally, condensation of ammonia with *N*-acylaminothiophenes **44** allowed access to 3-unsubstituted thieno[2,3-*d*]pyrimidin-4-ones **45** [15,17]. The first synthetic route involved 25% ammonia heated at 105 °C in a sealed vial to obtain thieno[3,2-*d*]pyrimidin-4-one **46a** after 3 h, with a 63% yield (Scheme 19). In contrast, using milder conditions with 30% ammonia at room temperature for 6 to 8 h led generally to lower yields (28–60%). Moreover, it has been observed by Desroches et al. that this method was not efficient when R = CCl_3_ (compound **45e**) [17]. Indeed, with this substrate, cyclization in the presence of 25% ammonium hydroxide in a sealed vial failed.

### 2.2. Synthesis of Thienopyrimidines from Pyrimidine Derivatives

#### 2.2.1. Synthesis from the Thorpe-Ziegler Reaction

**Scheme 20:**
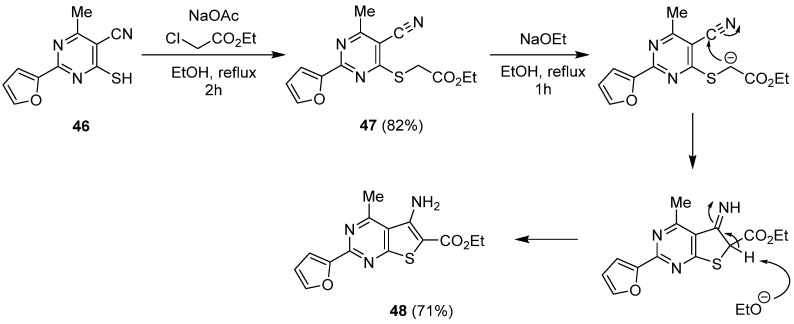
Synthesis of thienopyrimidin-4-one **48** via a Thorpe-Ziegler cyclization.

One of the possibilities to shape the thieno-fused ring from pyrimidine derivatives is the Thorpe-Ziegler cyclization. A six-membered ring bearing a mercaptocarbonitrile group was the starting point to synthesize thienopyrimidines (Scheme 20). After substitution of alkyl chloroacetate by the sulfhydryl group (compound **47**), and subsequent deprotonation, cyclization can occur in basic conditions. In such a way, Abdel Hamid et al. reported the synthesis of compound **48** with a 71% yield [38].

**Scheme 21:**
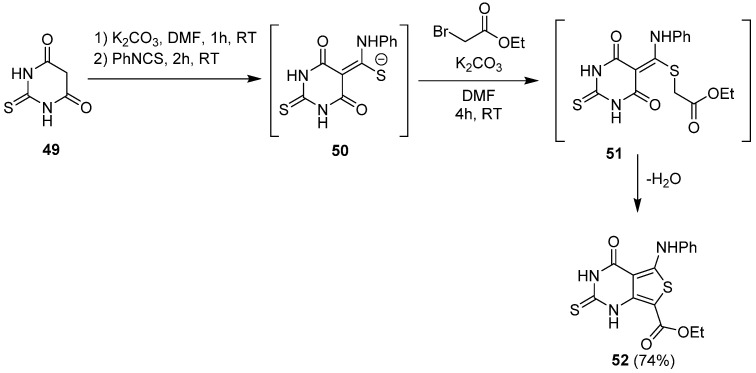
Synthesis of 2-thioxo-1,2,3,4-tetrahydro thieno[3,4-*d*]pyrimidin-4-one **52**.

A variant of the previous approach was purposed by Ali and Saleh for the synthesis of 2-thioxo-1,2,3,4-tetrahydrothieno[3,4-*d*]pyrimidine **52** [39]. First, thiobarbituric acid **49** was deprotonated in α-position of the two carbonyl groups at room temperature (Scheme 21). Then, nucleophilic substitution on phenyl isothiocyanate led to the ketene aminothioacetal **50**. Thereafter, the addition of alkyl bromoacetate allowed cyclocondensation of **51** in basic conditions. The final product **52** was obtained with good yields (74%).

#### 2.2.2. Synthesis from the Gewald Reaction

**Scheme 22:**
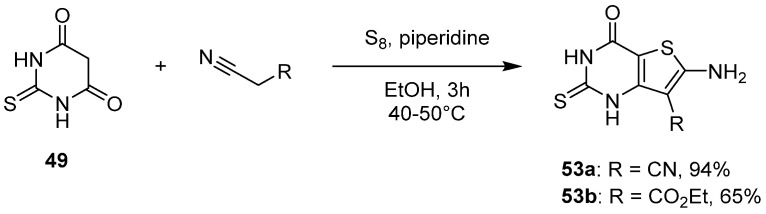
Synthesis of 2-thioxo-thieno[3,2-*d*]pyrimidines **53** by the Gewald reaction.

The Gewald reaction is a versatile reaction to access 2-aminothiophene derivatives involving one-pot cyclocondensation of ketones or aldehydes with activated nitrile derivatives and elemental sulfur. Using thiobarbituric acid **49** as the starting ketone, 2-thioxo-6-aminothieno[3,2-*d*]pyrimidin-4-one derivatives could be easily accessible. Treatment of **49** with piperidine in the presence of the appropriate alkyl cyanide led to the aminothieno-fused derivatives **53a** and **53b** with good yields (Scheme 22) [39].

As shown in the previous examples, many access routes to these compounds are possible and allow to easily prepare a wide range of polysubstituted thienopyrimidines. Therefore, these compounds have been included in many biological studies. More particularly, their antiparasitic, antibacterial, antifungal and antiviral activities have been studied.

## 3. Antiparasitic Activity of Thienopyrimidines

### 3.1. Antimalarial Activity

Malaria is a parasitic disease caused by protozoan parasites belonging to the *Plasmodium* genus. Five species are known to infect humans, namely *P. falciparum*, *P. vivax*, *P. malaria*, *P. ovale*, and *P. knowlesi*. The female *Anopheles* mosquito acts as the transmission vector of the infection. *P. falciparum* is the most virulent species in humans while *P. falciparum* and *P. vivax* represent the greatest threat [40]. In 2019, *P. falciparum* generated most malaria cases in Africa, South-East Asia, Eastern Mediterranean, and Western Pacific while *P. vivax* was the most prevalent in the Americas. In 2019, the number of cases was estimated at 229 million in the world and 409,000 deaths due to malaria were identified by the World Health Organization (WHO) [41]. The resurgence of resistance to current antimalarial drugs such as artemisinin derivatives [42] represents a major health issue. Therefore, the development of novel antimalarial drugs remains an urgent need [43].

#### 3.1.1. Thieno[2,3-*d*]pyrimidine Derivatives with Antiplasmodial Activity

Zhu et al. elaborated a small library of thieno[2,3-*d*]pyrimidine derivatives as falcipain-2 inhibitors [44]. The cysteine protease falcipain-2 (FP-2) of *P. falciparum* is a major cysteine protease and an essential hemoglobinase of erythrocytic trophozoites [45]. Inhibition of FP-2 blocks hemoglobin hydrolysis and stops the development of the parasite. Therefore, the FP-2 enzyme would represent an attractive target for antimalarial drug development [46]. Enzyme inhibition assays showed inhibitory potential for the whole series. The inhibition rate of these derivatives ranges between 53.0 and 94.3% at 10 μM (Table 1). Falcipain-2 inhibitors described in the literature are peptidic analogues that exhibit nanomolar IC_50_ values [47]. In contrast, Zhu et al. derivatives demonstrated moderate activity with micromolar IC_50_ values. IC_50_ values of these compounds against FP-2 showed that allyl, cyclohexyl, *para*- or *meta*-phenyl groups at position 3 were tolerated (**54a** to **54d**, IC_50_ = 1.46 to 2.81 μM). *Para*-chloro-phenyl and benzyl groups led to a slight loss of potency (**54e** and **54f**, IC_50_ = 4.30 and 5.74 μM, respectively). Replacing the phenyl group on the thiophene ring of **54a** by a *meta*-substituted phenyl group led to a loss of potency (**54h**), whereas a *para*-substituted phenyl group maintained activity (**54g**). *N*-substitution of the amide at position 2 globally maintained the inhibitory activity of compounds **55a** to **55f** with IC_50_ values from 2.49 to 6.63 μM.

Then, a series of thieno[2,3-*d*]pyrimidines was discovered by Edlin and Barrows’ teams as potent antimalarial agents with micromolar or submicromolar activities [48,49]. All derivatives synthesized were evaluated in vitro against *P. falciparum* NF54 or *P. falciparum* 3D7 (clone of NF54 and chloroquine-sensitive) strains (Table 2). SAR study demonstrated that bulky groups supported by the triatrazole ring at position 4 increased antiplasmodial activity (compounds **56a** and **56c** compared to **56b**). However, the introduction of methyl groups at R_1_ and R_2_ slightly decreased activity (**56c** vs. **56d**). Thereafter, a thiazole group linked with thioether at position 4 led to strong antiplasmodial activity (**56e**, EC_50_ = 34 nM on *P. falciparum* 3D7 strains). In contrast, a benzimidazole group reduced activity (**56f**, EC_50_ = 0.191 µM). Bioisosteres of **56e** and **56f** (**56g** and **56h**) were synthesized and evaluated to avoid the S-oxidation metabolism of the sulfur at position 4. Unfortunately, **56g** and **56h** were inactive against *P. falciparum* 3D7 strains. Only cytotoxicity of compounds **56a** to **56d** was determined (EC_50_ < 4 µM on HEK-293 mammalian cell lines). Afterward, **56c** was assessed in vivo on mice infected by *Plasmodium berghei* at 50 mg/kg once a day for 4 days. At the end of the experiment, the parasitemia decreased by 34%, and no gain in survival days was observed compared to the untreated mice.

Additional studies carried out with compound **56e** demonstrated that this family of compounds targets coenzyme A (CoA) synthetic pathway. CoA is involved in metabolic functions and is necessary for parasite survival. CoA operates during the asexual and sexual stages of *P. falciparum* [50,51,52]. Compound **56e** exhibited strong antiplasmodial activity on both the asexual and the sexual stage of *P. falciparum* (EC_50_s = 0.06 to 0.120 µM). **56e** could block parasite transmission. In addition, no cytotoxicity on HEK-293 cell lines was observed (EC_50_ = 40 µM) [50,51]. Then, Weidner et al. further studied this series of 4-thioether-thieno[2,3-*d*]pyrimidines [52]. To identify active compounds, a first screening was realized at 3 µM on the asexual erythrocytic stage of transgenic NF54-luc *P. falciparum*, using a luciferase-based viability assay. All derivatives presented in Table 3 decreased the viability of erythrocytic asexual stage *P. falciparum* NF54-luc parasites (compounds **56i** to **56o**). Then, the authors demonstrated that supplementation of CoA in the growing medium decreases the antiplasmodial activity of thienopyrimidines (*Pf*3D7 EC_50_ vs. *Pf*3D7 + 0.8 mM CoA EC_50_). These results corroborated the hypothesis that the inhibition of CoA synthesis was a mechanism associated with the antiplasmodial activity of this series. Lastly, all derivatives exhibited low to moderate cytotoxicity on THP-1 cells, a human monocytic cell line, and on HEK-293 cell line.

#### 3.1.2. Thieno[3,2-*d*]pyrimidine Derivatives with Antiplasmodial Activity

**Figure 2:**
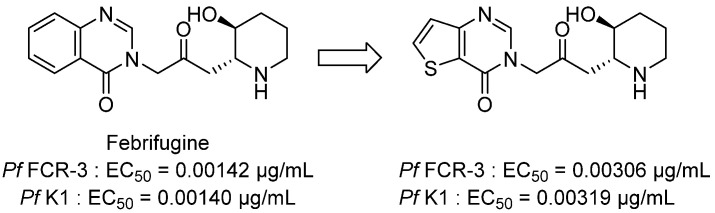
Structure and activity of a thienopyrimidinyl analogue of febrifugine.

In 2006, Kikuchi et al. synthesized a bioisostere of febrifugine represented in Figure 2 [53]. Febrifugine is a quinazolinone alkaloid first isolated from the Chinese herb *Dichroa febrifuga* Lour. which has been used in traditional Chinese medicine for over 2000 years for the treatment of various diseases, including malaria. Its analogue exhibited a strong antiplasmodial activity against *P. falciparum* FCR-3 and K1 cell lines (chloroquine-sensitive or resistant strains). Unfortunately, this analogue was cytotoxic on mouse-L929 cells (EC_50_ = 0.563 μg/mL). In vivo, this thienopyrimidinone exhibited similar activity than chloroquine (ED_50_ = 2.95 and 2.53 mg/kg, respectively) and a moderate cytotoxicity (LD_50_ = 88 mg/kg).

A few years later, Cohen et al. reported the antimalarial activity of a series of thieno[3,2-*d*]pyrimidinone derivatives. A library of 120 derivatives was synthesized and tested in vitro on *P. falciparum* K1-resistant strain and HepG2 cell line [36]. SARs in this series revealed that a *tert*-butyl- or an isopropyl-amino group at position 2 was essential to obtain antimalarial activity (compared compounds **57a–b** to compound **57c**, Table 4), along with a phenyl ring at position 6 (compared compound **57d** to **57e**). *Para*-substitution of this phenyl ring increased activity on *P. falciparum* strains (compared compound **57a** to **57d** and **57f–g**), but only with a methyl group or a chlorine atom (**57a**, **57h** vs. **57i**). To improve the aqueous solubility of these derivatives, various salts were synthesized. Hydrochloric salt **57j** was identified as a lead compound with an antimalarial activity tenfold higher than chloroquine and with cytotoxicity like chloroquine. The advantage of compound **57j** was its activity on the erythrocytic stage of *P. falciparum* K1 strain and the liver stage of *P. yoelii* strain (EC_50_ = 35 nM). A preliminary in vivo evaluation revealed that **57j** reduced parasitemia by 45% compared to untreated infected mice, proving that its antiplasmodial activity was preserved in vivo. Bosson-Vanga et al. discovered **57j** displayed activity on *P. falciparum* at the three stages of the parasite cycle (erythrocytic, hepatic and sexual stages) and reduced transmission of the parasite in a mouse model [9]. However, the original mechanism of action of these compounds remains to be elucidated.

González Cabrera et al. were interested in similar derivatives but including an amine function at position 4 of the thienopyrimidine core [35,54]. A SAR study demonstrated the influence of various substituents at positions 2, 4, and 6 of the thieno[3,2-*d*]pyrimidine ring (Table 5). These derivatives displayed in vitro activity on both *P. falciparum* chloroquine-sensitive strains (NF54) and resistant strains to chloroquine, pyrimethamine, and proguanil (K1) with EC_50_ values in the submicromolar range (Table 5). At position 6, a phenyl group was essential for antimalarial activity (**58a** vs. **58b**). *Para*- or *meta*-monosubstitution and *meta*-*meta*- or *para*-*ortho*-disubstitution of this phenyl ring by electron-withdrawing groups were tolerated (**58c** to **58h**). Compounds **58c** and **58f** were 10-fold more potent than chloroquine, with EC_50_ = 19 nM and 17 nM vs. 194 nM, respectively. Morpholinophenyl derivative **58i** displayed strong antimalarial activities against both strains (EC_50_ of 12 and 7 nM, respectively). Unfortunately, this compound showed poor in vitro microsomal stability after incubation with human liver microsomes (half-life = 15 min). The most stable compound **58d** was chosen for in vivo studies on a *P. berghei* infected mouse model. Compound **58d** reduced parasitemia by more than 99.8% when administered orally (50 mg/kg once daily for 4 days). This administration scheme led to a mean survival time of 23 days, which is comparable to chloroquine at 4 × 30 mg/kg. The major drawback of this series was the metabolic weakness due to the dealkylation of positions 2 and 4, along with the inhibition of hERG channels, which could cause cardiac toxicity [55]. The SAR study was extended and the metabolic stability of these thieno[3,2-*d*]pyrimidine derivatives was explored. Various aminoalkyl chains were introduced at position 4 (compounds **58j** to **58p**). These modifications led globally to a decrease in the activity, except when an aminopiperidine group or an alkylmorpholine group was introduced (compounds **58n** to **58p**). Interestingly, these latter compounds showed improved microsomal metabolic stability. Substitution of position 2 was also studied. Replacing the amino group with another substituent like a hydrogen or a chlorine atom, a methoxy, a morpholino or a phenylmethanesulfonyl group was not tolerated. In contrast, a benzylamino group (compound **58q**) or a primary amine (compounds **58r** to **58t**) was well tolerated. Compound **58r** presented good in vitro antimalarial activity and led to the *N*-methyl dealkylated as one of the main identified metabolites. Two new compounds, **58s** and **58t**, were identified as displaying a strong activity, 5- to 8-fold better than chloroquine on *P. falciparum* K1 and NF54 strains, together with a high in vitro microsomal stability. Unfortunately, these two thieno[3,2-*d*]pyrimidine derivatives exhibited significant affinity for hERG channels. The cytotoxicity of compounds **58n**, **58o** and **58t** was measured against CHO and Vero cell lines and evidenced a strong in vitro cytotoxic effect (CC_50_s between 2.17 and 4.30 µM).

**Figure 3:**
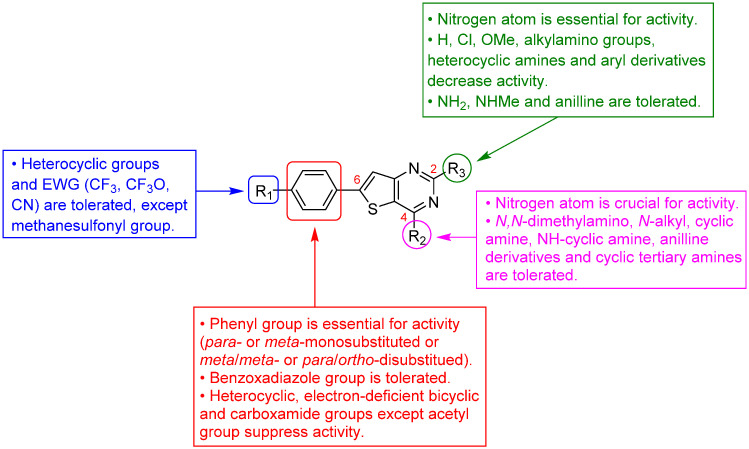
SARs of thieno[3,2-*d*]pyrimidine compounds according to González Cabrera and Van der Watt works.

Van der Watt et al. also highlighted the antiplasmodial activity of 2,4-diaminothienopyrimidine derivatives on the asexual blood stage of *P. falciparum* with nanomolar EC_50_s [56]. Twelve 2,4-diaminothienopyrimidines were studied on the asexual and sexual stages. Overall, these compounds presented a better activity on the asexual stage than on the sexual stage (gametocytes) of the parasite. These results are quite mitigated because the ultimate goal of antimalarial chemotherapy is to act on both the asexual and the sexual stage of the parasite, to eradicate its development in humans and block the transmission of the disease. Nevertheless, these thieno[3,2-*d*]pyrimidine derivatives were still slightly effective against early and late-stage gametocytes. SARs were inferred from the chemical features and biological activities in this series. A diaminothienopyrimidine scaffold, a *N*-methylation at position 2, and a *para*-phenyl substitution with lipophilic groups at position 6 were identified as important criteria for gametocidal activity (Figure 3).

Woodring et al. carried out a large repositioning campaign of compounds, by screening them against several protozoan parasites [14,57]. Some of them incorporating a thienopyrimidine core and a fluorobenzylaminophenoxy group were identified as potential antimalarial drugs. These derivatives presented EC_50_ values in the order of the micro- or submicromolar range on drug-sensitive D6 *P. falciparum* strains (Table 6). SAR studies revealed that morpholinophenyl (compounds **59a** and **59b**) and alkynyl substituents (**59c** and **59d**) at position 2 led to very good antimalarial activity. A lead compound **59a** was identified as displaying a strong in vitro antimalarial potency against *P. falciparum* sensitive D6 strains (EC_50_ = 27 nM).

Further works, carried out by Desroches et al., also demonstrated that compounds with a thienopyrimidine core, in [3,2-*d*] or [2,3-*d*] series, exhibited antimalarial activities on K1 *P. falciparum* resistant strains with EC_50_ values in the submicromolar range [17]. Substitution of positions 2 and 4 was explored exclusively. SAR studies at position 2 were performed on the pyrimidine core. Various substituents were introduced such as a hydrogen atom, a methyl, or halogenated methyl groups. The antiplasmodial activity was maintained only in the presence of the trichloromethyl group. Despite the promising in vitro activity profile of these compounds, consequent cytotoxicity on HepG2 human hepatic cell line and low selectivity were the main drawbacks of these series (Table 7). Interestingly, the SAR study evidenced no significant difference between the two thienopyrimidine series.

Through the various examples presented above, the thienopyrimidine core proved to be attractive to discover new antimalarial agents. In these different studies, thieno[2,3-*d*]pyrimidines and thieno[3,2-*d*]pyrimidines were considered and modulations at positions 2, 3, 4, 5, 6 and 7 of the thienopyrimidine core have been carried out to reach antiplasmodial activities in the order of the micro or submicromolar range. The most promising compounds seem to be thieno[3,2-*d*]pyrimidines substituted in positions 2, 4, and 6. Their weak point remains their significant cytotoxicity on HepG2 and CHO cells. In addition, for most of the examples presented, no target was identified except for compounds **54** and **55,** which inhibited the cysteine protease falcipain-2 (FP-2). Further works need to be carried out to identify the plasmodial target(s) involved in the mechanism of other compounds.

### 3.2. Anti-Trypanosomatid Activity

Trypanosomatid parasites are the causative agents of several neglected tropical diseases [58]. Among them, *Trypanosoma brucei*, *Trypanosoma cruzi*, and *Leishmania* sp. are responsible for human African trypanosomiasis (HAT), Chagas disease, and leishmaniasis, respectively. Regarding leishmaniasis, *L. donovani* and *L. infantum* cause visceral leishmaniasis in humans, which is a mortal disease if untreated.

In 2015, after having tested thienopyrimidines on *P. falciparum* strains [14] (see previous section), Woodring et al. screened more than 35 of these compounds on *L. major* and identified several molecules with micromolar activity on the amastigote form and submicromolar activity on the promastigote form of the parasite (Table 8). To broaden the spectrum of anti-trypanosomatid activities, a new thienopyrimidine scaffold was designed. It was inspired by compound GW837016X [59] (compound **59a**), which presented good activity on *T. brucei* strains with an EC_50_ = 0.26 μM. Compound **59b** was the most potent molecule of this series against *L. major* promastigotes (EC_50_ = 0.22 µM) but displayed no activity on the amastigote form (EC_50_ > 15 µM), making it irrelevant for potential human use (Table 8). To explore their whole antitrypanosomatid potential, Woodring et al. also evaluated these compounds on *T. brucei brucei* strain 427 and *T. cruzi* amastigotes. A single compound, **59c**, presented a strong activity against *T. b. brucei*, with submicromolar activity comparable to the reference drug suramin, and no cytotoxic effects toward the HepG2 cell line. **59d** was also identified as a potent anti-*T. cruzi* hit compound, with the same in vitro activity as benznidazole. Three years later, new thieno[3,2-*d*]pyrimidine derivatives substituted by various alkynyl groups at position 6 were synthesized by the same team [57]. Among these compounds, **60b** revealed a strong activity against *T. cruzi* amastigotes, like benznidazole. Only **59e** presented a submicromolar EC_50_ value against *L. major* amastigote, but with an activity 10-fold lower than amphotericin B. Other compounds **59f**, **59g** and **60a** displayed antitrypanosomal activity on *T. b. brucei* with submicromolar EC_50_s. Complementary experiments proved that **59a** stopped trypanosome proliferation after G2 phase and before cytokinesis.

**Figure 4:**
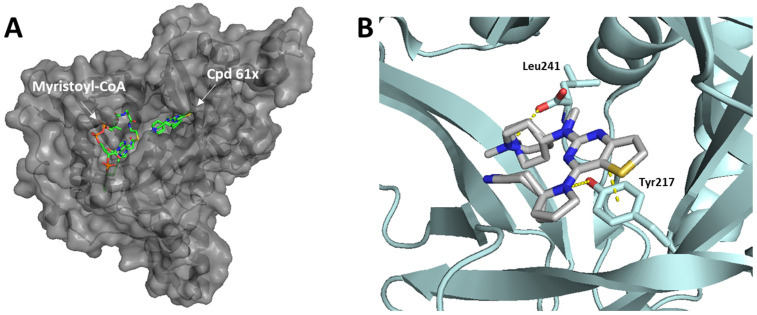
Co-crystallized structure of compound **61x** and *L. major* NMT (PDB: 6QDD). (**A**)—Positioning of **61x** and myristoyl-Co A in the pockets of NMT. The surface of the enzyme is represented in gray and the compounds are shown as stick representations. (**B**)—Key interactions of **61x** into the active site of NMT. The enzyme is represented as pale cyan cartoon mode and the compounds are shown as stick representation. Key bonding interactions are indicated as yellow dotted lines.

Five years later, Bell et al. developed novel thienopyrimidines as inhibitors of leishmanial *N*-myristoyltransferase (NMT) [60]. NMT plays a key role in the growth and development of eukaryotes by catalyzing the co-translational *N*-terminal myristoylation of several proteins. Bell et al. studied thienopyrimidine derivatives, substituted at positions 2, 4, and 6 (Table 9). Position 4 was functionalized by an aminopropionitrile group and modifications at other positions were explored. Compounds **61a** and **61b** were identified as potential selective inhibitors of NMT in *L. donovani* (IC_50_ = 0.34 µM and 0.15 µM, respectively) and *L. major* NMT (IC_50_ = 0.20 µM and 2.7 µM, respectively). Replacing the piperidine ring with a tetrahydropyran (**61c**), a hydroxypropyl (**61d**), a piperazine (**61e–f**), cyclic amines (**61g**), or a pyrrolidine-amine (**61h**) led to a loss of potency (IC_50_ from 7.7 µM to >100 µM). Slight modifications on this piperidine substituent at position 2 (**61i–j**) were tolerated, except when a ramified alkyl chain was introduced (compound **61k**). Modifying the diamine chain of compound **61b** caused a decrease in the inhibitory activity on *L. donovani* NMT (**61l–n**). Replacement of the aminopropionitrile group at position 4 by a hydrogen atom, alkyl ether (**61p**), amino alcohol (**61q**), amino amide (**61r–s**), alkyl amine (**61t**), and pyrrolidine group (**61u**) led to a drop in potency on *L. donovani* and *L. major* NMT. However, compound **61v**, substituted by a pyrrolidine group at position 4 and an *N*-methylpiperidine group at position 2, exhibited a submicromolar activity against *Ld*NMT and a strong selectivity for leishmanial NMT over the human isoform (IC_50_(*Hs*NMT1)/IC_50_(*Ld*NMT) > 660). Moreover, a *tert*-butyl group at position 6 (**61w** and **61x**) improved the *Leishmania* NMT inhibition. Compound **61x** exhibited the best IC_50_ value against *L. donovani* and *L. major* NMT (46 and 42 nM, respectively). However, the selectivity against the human form of the enzyme was lost. The co-crystallization of 12 derivatives complexed with *L. major* NMT and myristoyl-CoA highlighted the key inhibitor-enzyme interactions. Co-crystallization of compound **61x** suggested that the aromatic system of the pyrimidine core was involved in π-π staking interactions with Tyr217. The lipophilicity of the rigid core and the specific geometry of the piperidine group at position 2 appeared to be crucial interaction parameters. Moreover, the basic center of this group established an ionic interaction with the carboxylate of the *C*-terminal Leu421 carboxylate. Finally, the nitrogen atom of the pyrrolidine at position 4 was also essential as it formed a hydrogen bond with the hydroxyl group of Tyr217 (Figure 4).

### 3.3. Antihelminthic Activity

To our knowledge, only one study reported the activity of thienopyrimidine derivatives on helminths, and more specifically against *Trichinella spiralis*. Trichinellosis is caused by larva of these nematodes, which settles in the muscular tissues of the host. Humans get infected by this parasite after consumption of raw or inadequately cooked meat, containing encysted larvae.

Mavrova et al. synthesized and evaluated thieno[2,3-*d*]pyrimidine derivatives as antihelminthic agents against *Trichinella spiralis* [19]. The substitution of the alkyl chain in position 2 of the thienopyrimidine ring by a benzimidazole moiety was essential for the antihelminthic activity (Table 10). The most active compound **62a** presented after 48h incubation a percentage of efficacy against *T. spiralis* larvae 5-fold better than albendazole, chosen by the authors as the reference drug. The addition of a sulfide group at position 2, as a link between the ethyl chain and the benzimidazole ring, was also tolerated (compounds **63a** and **63c**, 59.75% and 80.05% efficacy after 48 h incubation, respectively), except for **63c** which was not active in vitro. Further experiments also demonstrated an in vivo antiprotozoal activity of these compounds against Lamblia muris.

## 4. Thienopyrimidines with Antituberculosis Activity

Tuberculosis (TB) is an infectious disease caused by the bacterium *Mycobacterium tuberculosis* (MTB). This mycobacterium spreads through the air and infects the lungs. The WHO reported that 10 million people contracted TB and approximately 1.5 million died from this infection in the world in 2020 [61]. The most vulnerable people to TB are those that already have a disease that weakens their immune system, such as human immunodeficiency viruses (HIV) infection. However, this infection remains curable, except for multi-drug-resistant TB (MDR-TB), which contaminated approximately 206,000 people in 2019 [61]. TB is the source of a public health crisis as a threat to health security. Nowadays, the major issue is to offer new antibacterial treatments, effective on MDR-TB, inexpensive and accessible to all.

In this context, Rashmi et al. discovered new potent antituberculosis agents with a thieno[2,3-*d*]pyrimidine core [62]. All synthesized compounds were evaluated against MTB H37Rv (AT27294) by determining minimum inhibitory concentration (MIC). Regarding the SAR study, electron-donating substituents of the phenyl group, in *para-* or *ortho*-position (compounds **64b–f**) led to higher antituberculosis activity compared to compound **64a** (MICs = 32 to 71 and 320 µM, respectively) and similar activity to pyrazinamide, used as the reference drug (MICs = 64 to 71 µM and 60.97 µM, respectively) (Table 11). Bulkier 3,4,5-trimethoxyphenyl group was also tolerated (compound **64g**). The most interesting compounds demonstrated weak cytotoxicity against THP-1 human monocytic cell line.

From a high-throughput screening of a 100,997 compound library, Ananthan et al. identified thienopyrimidinone derivatives with antituberculosis potential [63]. Five of these derivatives (compounds **65a–e**) exhibited moderate to high antimycobacterial activity against MTB H37Rv (Table 12). However, the limited number of thienopyrimidines in the screened libraries as well as the lack of reported IC_90_ for reference drugs included in the assay do not allow any firm conclusion on SARs.

Several years later, Harrison et al. reported the activity of a series of 4-amino substituted thieno[2,3-*d*]pyrimidine derivatives against *Mycobacterium tuberculosis* [64] (Table 13). Biological studies were carried out on *M. tuberculosis* strains to determine antibacterial activities via a Microplate Alamar Blue assay (MABA). Regarding SAR studies, some derivatives with a long aminoalkyl chain displayed significant antibacterial activities with IC_50_ values in the micromolar or submicromolar range (IC_50_ = 0.083 to 2.7 µM). A lead compound (**66f**) was identified with stronger antituberculosis activity compared to the reference drug thioridazine (IC_50_ = 0.083 and 11.2 µM, respectively) but with a similar activity than bedaquiline. Several bulky groups at R such as alkyl and alkylaryl chains were tolerated (**66b** to **66e** and **66g**). Various experiments carried out by Harrison et al. indicated that these 4-aminothieno[2,3-*d*]pyrimidines could target QcrB, a subunit of the electron transport chain (ETC) enzyme cytochrome bc1 oxidoreductase [64]. A recent study suggests that combination of QcrB inhibitors and current treatments tends to amplify the antimycobacterial activity of the treatments and presents a possible alternative to improve current antitubercular drugs [65]. However, the safety of such approach needs to be confirmed.

## 5. Thienopyrimidines with Antibacterial Activities Other than Tuberculosis

Since the discovery of the first antibiotic, penicillin G by Alexander Fleming in 1928, antibiotics have been extensively used to treat all types of microbial infections. However, despite the existence of a wide range of antibiotics, the number of bacterial infections is constantly increasing with greater difficulties to cure them [66]. Even if the reasoned use of antibiotics has limited the development of resistance, this strategy is not sufficient to stop its progression and bacterial resistance becomes a growing scourge for humans. Therefore, the development of new antibiotics became an urgent concern, and the scientific community is thus mobilized to find new efficient antibacterial candidates. Among the different scaffolds under study, thienopyrimidine derivatives revealed to be attractive to discover new antibacterial compounds. However, the identification of active compounds in these series was mostly performed by phenotypic screening. To our knowledge, only one study reports an activity on an identified target, namely an amino-sugar acetyltransferase enzyme, named protein glycosylation D (PglD).

### 5.1. Inhibition of the Protein Glycosylation D (PglD) of Campylobacter Jejuni

*Campylobacter jejuni* is an intestinal Gram-negative bacterium. It most often causes severe diarrhea, which can be fatal to young children. *C. jejuni* can also be the cause of other serious infections such as hepatitis, pancreatitis and could provoke miscarriages, autoimmune diseases, or Guillain–Barré syndrome [67]. In recent years, the emergence of resistant strains toward front-line antibiotics against this bacterium was increasingly observed [68]. It has been reported that highly modified sugars, including 2,4-diacetamido-2,4,6-trideoxy-D-glucose (2,4-diacetylbacillosamine or diNAcBac) play a key role in host-cell interactions and can influence the virulence of Gram-negative bacteria. In addition, when certain enzymes involved in carbohydrate biosynthesis are suppressed, bacterial strains lose their activity. In this context, De Schutter team discovered a series of thienopyrimidine derivatives as inhibitors of an amino-sugar acetyltransferase enzyme, named protein glycosylation D (PglD), essential in the biosynthesis pathway of UDP-2,4-diacetamidobacillosamine of *C. jejuni* [18].

A wide range of compounds was synthesized and evaluated in vitro on *C. jejuni* (NCTC 11168) PglD acetyltransferase. Optimization of these thieno[2,3-*d*]pyrimidines activity was established from compound **67a** (Table 14). Replacement of the methyl group by a bulkier group such as phenyl substituent doubled affinity for PglD (**67a** vs. **67b**). *Para*-phenyl substitution or di-substitution in position 4 led to a strong PglD inhibition with submicromolar IC_50_s values (compounds **67c** to **67f**). In addition, *ortho*- or *meta*-substitutions were tolerated. Other bulky substituents such as pyridin-2-yl (**67g**) and benzo[*d*][1,3]dioxol-5-yl (**67h**) increased activity. Then, the replacement of the phenyl group at position 2 by a pyridin-3-yl ring led to a strong PglD inhibition (**67i** vs. **67a**). Insertion of a 4-acetamidophenylethyl group (**67j**) in position 2 allowed good *C. jejuni* PglD inhibition, the same way as a 2-methoxy-2-phenylethyl group (**67k** to **67m**).

**Figure 5:**
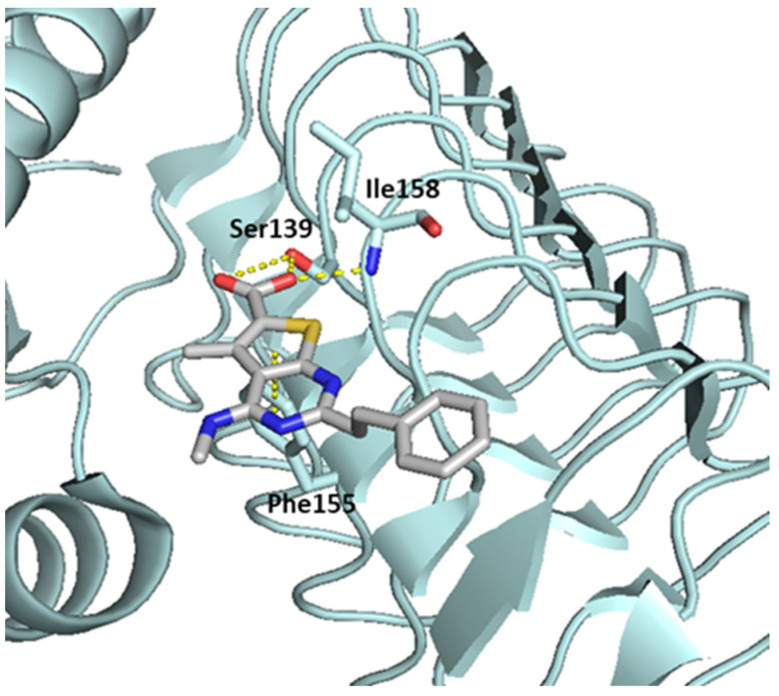
Co-crystallized structure of compound **67a** in the protein glycosylation D (PglD) active site (PDB: 5T2Y). The enzyme is represented as pale cyan cartoon mode and the inhibitor is shown as stick representation. Key bonding interactions are indicated as yellow dotted lines.

A co-crystallization of *C. jejuni* PglD with inhibitor **67a** was obtained (Figure 5) and three ligand–receptor interactions were identified. A π-staking interaction between the thiophene ring and Phe155 of PglD was observed. In addition, the carboxylic acid of inhibitor **67a** formed two hydrogen bonds with Ser139 and Ile158.

### 5.2. Compounds wih Broad-Spectrum Antibacterial Activity

In this section, the compounds were classified according to their chemical structure.

#### 5.2.1. Thieno[2,3-*d*]pyrimidin-4-one and Pyrimidin-2,4-dione Derivatives

Abu-Hashem et al. identified six thieno[2,3-*d*]pyrimidinone derivatives as antibacterial agents [31]. All final compounds were evaluated in vitro against *Escherichia coli*, *Staphylococcus aureus*, and *Bacillus cereus* (Table 15). Compounds **68** and **69a–c** displayed high activity against the three tested bacterial strains. Interestingly, their thienopyridine analogues **71a** and **71b** only showed moderate antibacterial activity, which could suggest that the carbonyl group at position 4 is important for the activity. Similar thienopyrimidin-4-ones **70a–b** and thienopyrimidine-2,4-diones **69d–e** were reported by Ortikov et al. Compounds were evaluated in vitro (Table 15) against the three previous bacterial strains and against *P. aeruginosa* [11]. Globally, this series showed lower antibacterial efficacy than the previous series, suggesting that the presence of a benzoyl group at R_1_ is important for the activity (compare **69c** to **69e**). Moreover, replacing the methyl group (**70a**) at R_2_ with a nitro group (**70b**) increased antibacterial activity against all tested strains. In addition, a sulfur atom in position 2 (**69e**) led to a better antibacterial activity than its oxo analogue **69d** against *S. aureus* and *B. cereus*. In contrast, **69d** strongly inhibited *E. coli* growth compared to **69e** (16 vs. 6 mm, respectively). However, none of these compounds exhibited better antibacterial activity than levofloxacin, used as the reference drug.

De Candia et al. studied a series of spiro thienopyrimidin-4-one derivatives against several resistant bacterial strains to usual antibiotics [69] (Table 16). These compounds were screened in vitro to determine their minimum inhibitory concentration (MIC). Globally, all compounds displayed lower antibacterial activity than ampicillin against the three tested strains (*S. agalactiae*, *E. faecalis* and *S. epidermidis)*. Regarding SAR studies, the modification of R_1_ did not affect the activity (compounds **72a** to **72c**). At R_2,_ the introduction of a phenyl group was tolerated (compound **72b**). However, the introduction of a *para*-methoxy (**72h**) or a *para*-nitro (**72g**) substituent on the phenyl ring decreased the activity, whereas a *para*-methoxy group maintained it (**72h**). Replacement of the methyl carboxylate (**72d**) by an acetyl group at position Y (**72e**) or replacement of the hydrogen atom (**72d**) by a methyl carboxylate group at position X (**72f**) were not tolerated. In addition, cytotoxicity of compounds **72b** and **72d** was determined on four cancer cell lines (Table 17). Compound **72b** revealed a moderate to strong cytotoxicity (GI_50_ = 8 to 23 µM). A slightly lower cytotoxic effect on cancerous cell lines was observed for compound **72d** (GI_50_ = 16 to 48 µM).

A wide range of thienopyrimidinone derivatives, substituted in positions 2, 3, 5, and 6, were synthesized by Shaaban et al. and Habib et al. [12,33]. Antibacterial activity was determined against a large panel of Gram-positive and Gram-negative bacteria. However, all tested compounds were revealed to have weaker antibacterial activities than the reference drugs (Table 18). In particular, compounds **73b**, **73e**, **73g–h** displayed moderate antibacterial activity against *B. subtilis*, 2-fold lower than ampicillin (MIC = 25 vs. 12.5 µg/mL, respectively). Regarding inhibition of Gram-negative bacteria, compounds **73b–c**, **73e**, **73g–h** were half as potent as levofloxacin against *P. aeruginosa* (MIC = 25 vs. 12.5 µg/mL, respectively). In the same way, *P. vulgaris* was modestly inhibited by compounds **73a**, **73d**, **73f**, and **73i** (MIC = 25 µg/mL).

Chambhare et al. studied a series of 5-furyl-thienopyrimidone derivatives that were tested in vitro against Gram-positive (*Staphylococcus aureus*, *Bacillus subtilis*) and Gram-negative bacteria (*Escherichia coli* and *Salmonella typhi*) [37]. Among these molecules, twelve of them demonstrated strong antibacterial activity against the four tested strains. *Para*-substitution of the phenyl ring with electron-donating groups associated with a carboxamide or an NCH spacer was associated to good antibacterial activities (compounds **74a–b**, **74e–f**) (Table 19). Nonetheless, compounds **74c** and **74g** bearing a *para*-nitro group exhibited even stronger antibacterial activities (MIC = 8 to 12 µmol·L^−1^ and 4 to 7 µmol·L^−1^, respectively). *Ortho-para*-halogenation also increased antibacterial activity against all bacterial strains (**74d** and **74h** compared to **74a** and **74e**). Overall, this series represented a broad-spectrum antibacterial potential.

**Figure 6:**
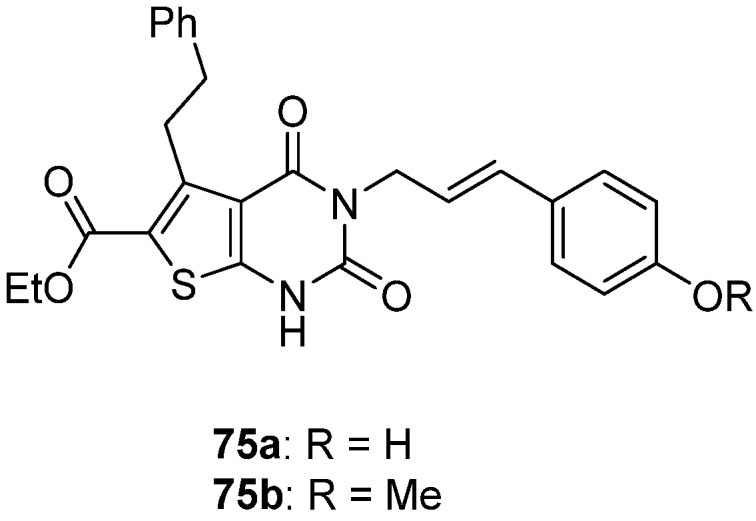
Structure of two thieno[2,3-*d*]pyrimidin-2,4-dione derivatives as antibacterial candidates identified by Dewal et al.

Two derivatives were identified as antibacterial agents by Dewal et al. [32] (Figure 6). Compound **75a** displayed broad-spectrum antibacterial properties with MIC values comprised between 2 and 32 mg·L^−1^ against vancomycin-resistant *S. aureus*, *S. pneumoniæ*, *E. faecium*, *P. aeruginosa*, *K. pneumoniæ*, and *E. aerogenes*. In contrast, compound **75b** only inhibited *E. aerogenes* with MIC equal to 8 mg·L^−1^. Compounds **75a–b** showed a slight cytotoxic effect against NIH-3T3 mammalian cells (GI_50_ = 52 and 98 mg·L^−1^, respectively). In addition, compound **75a** had no hemolytic activity.

#### 5.2.2. Other thieno[2,3-*d*]pyrimidine Derivatives

Tolba et al. discovered a novel series of thieno[2,3-*d*]pyrimidine derivatives with antibacterial potential [70]. Four compounds were synthesized and evaluated in vitro against *Bacillus cereus*, *Staphylococcus aureus*, *Pseudomonas aeruginosa*, and *Escherichia coli* (Table 20). All derivatives were active against these bacterial strains at low concentrations (MICs = 4.0 to 5.0 µg·mL^−1^), except compound **76d** on *E. coli* (MIC = 8.0 µg·mL^−1^). All these compounds showed antibacterial activity in the same range as reference drugs.

Saddik et al. identified new antibacterial candidates with a thienopyrimidine core substituted in positions 2, 4, 5, and 6 [71]. All compounds were evaluated against five bacterial strains (Table 21) and molecules **77g** and **77i** were the only derivatives showing moderate activity against *Staphylococcus aureus* and *Escherichia coli*, respectively. Regarding the SAR study, benzoyl (compound **77d**) and carboxamide groups (compounds **77b**, **77f–i**) at position 6 (R) were not tolerated against *Salmonella* sp. In contrast, cyano (compound **77a**), ethyl carboxylate (compound **77c**), and acetyl groups (compound **77e**) led to moderate activity against *Salmonella* sp. All derivatives were slightly active against *Bacillus subtilis* compared to the reference drug ampicillin, except compounds **77c**, **77g**, and **77i**.

Abdel Hamid et al. discovered a new thieno[2,3-*d*]pyrimidine series substituted in positions 2, 4, 5, and 6 [38]. Antibacterial activity of these derivatives was determined by measuring the inhibition zone diameter of Gram-positive and Gram-negative bacterial growth (Table 22). All derivatives showed moderate antibacterial activity against the tested strains. Regarding the SAR study, modification of ethyl carboxylate (**78a**) at R_2_ by carboxamide groups (**78b** and **78c**) implied similar potency. In the same way, modification of the amine at R_1_ by *N*-heterocyclic ring (**78d** and **78e**) and thiourea (**78f**) led to equivalent antibacterial activities. Only compound **78g** demonstrated similar antibacterial activity against *B. subtilis*, *E. coli*, and *P. aeruginosa* compared to ampicillin.

New thieno[2,3-*d*]pyrimidine derivatives incorporating an aminophenyl group or a pyrazole ring at position 4 were identified by Bhagchand et al. and Prabhakar et al. for their antibacterial potential [27,72]. Seven compounds were evaluated in vitro to determine the zone of growth inhibition of *Escherichia coli*, *Bacillus sphaericus*, *Bacillus subtilis*, *Staphylococcus aureus*, and *Klebsiella pneumonia* (Table 23). All derivatives exhibited a moderate activity against *E. coli*, whereas only compounds **79a** and **79b** were slightly active against *B. sphaericus*. However, the main drawback of this study was the absence of reference drugs. In contrast, other bacterial strains such as *B. subtilis*, *S. aureus*, and *K. pneumonia* were moderately inhibited by compounds **79d** to **79g** compared to amoxicillin.

**Figure 7:**
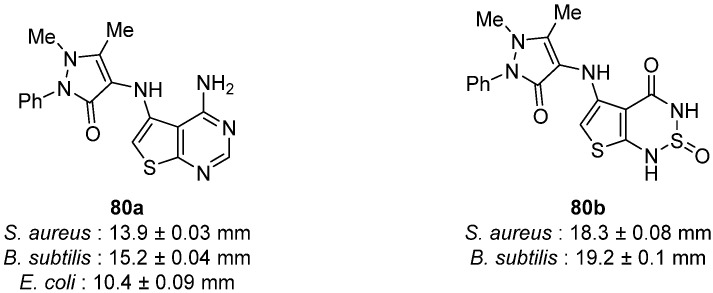
Antibacterial agents proposed by Aly et al. [16].

Aly et al. studied a thieno[2,3-*d*]pyrimidine derivative **80a** including an aminopyrazole at position 5 [16], as well as its original thienothiadiazine analogue **80b**. These two compounds were evaluated against *Staphylococcus aureus*, *Bacillus subtilis*, and *Escherichia coli* by determining a zone of growth inhibition of bacteria. Compounds **80a** and **80b** exhibited moderate antibacterial activity (Figure 7).

Afterward, new antibacterial agents were suggested by Kanawade et al. [13]. A series of sixteen derivatives was synthesized and evaluated in vitro against Gram-negative (*Escherichia coli* and *Pseudomonas aeruginosa*) and Gram-positive bacteria (*Staphylococcus aureus* and *Streptococcus pyogenes*). Overall, these compounds demonstrated weak antibacterial activities compared to the reference drugs tested in the same conditions (Table 24).

#### 5.2.3. Thieno[3,2-*d*]pyrimidine Derivatives

**Figure 8:**
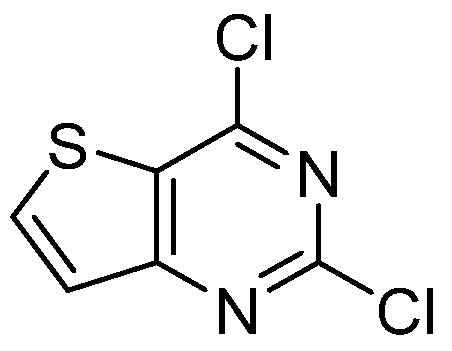
Structure of 2,4-dichlorothieno[3,2-*d*]pyrimidine.

Temburnikar et al. synthesized three 2-chlorothieno[3,2-*d*]pyrimidine derivatives [25] and evaluated them against several bacterial strains, including resistant strains (*Escherichia coli*, *Bacillus subtilis*, methicillin-resistant *Staphylococcus aureus*, vancomycin-resistant *Enterococcus faecalis*, and *Pseudomonas aeruginosa*), and also against several fungi (see Section 6). Among these three compounds, only 2,4-dichlorothieno[3,2-*d*]pyrimidine (Figure 8) showed a low antibacterial activity against one bacterial strain, *B. subtilis* with 43% inhibition at 100 µM.

Giri et al. identified new thieno[3,2-*d*]pyrimidine derivatives including an acyl hydrazone moiety as potential antibacterial agents [23]. All derivatives were evaluated in vitro against *E. coli*, *Pseudomonas* sp., *S. aureus*, and *Bacillus* sp. (Table 25). In this series, modulations were only studied on the hydrazone part of the molecule. Globally, these compounds showed similar activities against the tested strains, except compounds **83c** and **83f**, which were only active towards *S. aureus* and *Pseudomonas*, respectively. However, the exhibited antibacterial activities were twice lower than the reference drug streptomycin.

Afterward, Hafez et al. undertook considerable work on spiro derivatives of thieno[3,2-*d*]pyrimidine-4-one [73]. Approximately forty compounds were synthesized and evaluated in vitro on six Gram-positive and Gram-negative bacterial strains (Table 26). Regarding SAR studies, the introduction of an oxygen atom in position 3 (compound **84a**) caused a loss of potency compared to compounds **84b–j**. Compounds **84b**, **84e**, **84h–j** displayed better or similar inhibitory activity against all bacterial strains (MIC = 1 to 4 µmol·L^−1^) than the reference drug ciprofloxacin. In contrast, compounds **84c–g** exhibited moderate antibacterial activity. Overall, all these compounds appeared to be broad-spectrum antibacterial molecules.

Shao et al. also reported the antibacterial activity of a series of thieno[3,2-*d*]pyrimidine-4-one [15], showing that such compounds may be effective against *Clostridium difficile*. This Gram-positive bacterium is naturally present in the intestinal flora of humans. In frail people, taking some antibiotics such as amoxicillin, clindamycin, and cephalosporins may cause *C. difficile* infection, which can be difficult to treat [74]. During this infection, bacterial toxins formation can lead to severe diarrhea and pseudomembranous colitis [75]. A wide range of 67 thieno[3,2-*d*]pyrimidin-4-one incorporating a nitro group at position 7 was synthesized and evaluated against two *C. difficile* strains (ATCC BAA 1870 and ATCC 43255). A dozen of these molecules inhibited *C. difficile* moderately, with MIC values between 1 and 16 µg·mL^−1^. Regarding the SAR study, positions 2, 3, and 4 were modified. Small groups such as a hydrogen atom or a methyl group at R_1_ led to moderate antibacterial activity (**85a** and **85b**, Table 27). The introduction of a styryl group (**85c**), its bioisostere vinylthiophene (**85d**), or a *meta*-substituted phenyl ring (**85g**) kept the activity. In contrast, *ortho*- and *para*-phenyl substitution slightly increased antibacterial activity (**85e–f**, **85h–I** compared to **85c**) on *C. difficile*. Other positions were then modulated while keeping a methyl group in position 2. Globally, benzyl substituents at position 3 were tolerated (**85j–k**); the same holds for the insertion of a chlorine atom at position 4 (**85l**). Unfortunately, none of these derivatives displayed equal or higher activities compared to the three reference drugs. Finally, all derivatives exhibited low cytotoxicity against human colorectal adenocarcinoma (Caco-2), human ileocecal adenocarcinoma (HRT-18), and African green monkey kidney cells (Vero) and were inactive on Human Normal Microflora.

Finally, Aly and Saleh synthesized two thieno[3,2-*d*]pyrimidine derivatives **53a–b** and one isomer **52** [39] that were screened on two bacterial strains, namely *P. aeruginosa* and *S. aureus*. These compounds inhibited moderately these bacteria compared to tetracyclin, chosen by the authors as the reference drug (Table 28).

## 6. Thienopyrimidines with Antifungal Activity

Nowadays, fungal infections continue to develop in humans and can lead to serious complications in patients with comorbidities causing immunosuppression (acquired immunodeficiency syndrome (AIDS), asthma, cancer, organ transplantation, corticosteroid therapies). The development of medico-surgical practices increases partly the risk of fungal infections. Fungal infections remain a growing scourge, with 150 million severe cases each year and approximately 1.7 million deaths per year worldwide [76]. In the same way as the overuse of antibiotic therapies, the escalating use of antifungals has led to the emergence of multi-drug-resistant fungi. The most threatening fungal pathogens are *Aspergillus* sp. and *Candida* sp., especially *Candida auris*, because of their resistance to most of currently available treatments [76]. To date, three main classes of antimycotic drugs are marketed, namely azoles, echinocandins and polyenes. As a large range of thienopyrimidines could display antibacterial activity (see Section 5), some studies have also evaluated the activity of such compounds on various fungal strains. In this section, only compounds showing antifungal activity at least similar or quite similar to the reference drug included in the study have been reported.

Abu-Hashem et al. evaluated their thienopyrimidin-4-one **68** and 2,4-diones **69** against *Candida albicans* and *Aspergillus niger*, by determining an inhibition zone diameter of growth [31]. All derivatives showed quite similar antifungal activity to nystatin. Hydrogen, phenyl, or amine groups in position 3 were well tolerated and led to similar activity on the two fungi, compared to the reference drug (**68**, **69a**, and **69c**, Table 29). In addition, replacement of the amino group at position 2 by a sulfur or oxygen atom maintained antifungal activity (**68** vs. **69a** to **69c**). In contrast, replacing the thienopyrimidine moiety with a thienopyridine core caused a loss of potency (**71a** and **71b**), as also observed previously for their antibacterial activity.

Aly and Saleh also tested their 2-thioxothienopyrimidin-4-(3*H*)-ones against two fungal strains, namely *Aspergillus flavus* and *Candida albicans* [39]. Compound **55** was inactive against *A. flavus* and 2-fold less active on *A. niger* compared to amphotericin B (Table 30). In contrast, compounds **53a** and **53b** showed similar activity to amphotericin B, although slightly lower.

Hafez et al. evaluated their antibacterial thieno[3,2-*d*]pyrimidines series (see previous section) against *Candida albicans* (ATCC 15056), *Aspergillus flavus* (ATCC 24556), and *Ganoderma lucidum* (ATCC 96918) [73]. Among the tested compounds, seven showed high antifungal activity against the three fungal strains, with MICs from 1 to 7 µmol·mL^−1^ (Table 31), similar to ketoconazole used as the reference drug. Regarding SAR study, a *para*-halogenophenyl or a 2-thienyl group (**84k** to **84m**) linked to the pyrazolyl moiety allowed strong antifungal activity (MICs = 1 to 5 µmol·mL^−1^). Introduction of several heterocyclic substituents at position R was tolerated. Introduction of an isoxazolyl group (**84n**) slightly decreased the growth inhibition of the three fungal strains (MICs = 4 to 7 µmol·mL^−1^), whereas the presence of a thioxopyrimidinyl group (**84o–q**) resulted in the retention of antifungal activity, with a slightly lower activity than the reference drug ketoconazole (MICs = 3 to 5 vs. 2 to 3 µmol·mL^−1^).

In the same way, Shaaban et al. have also studied their thieno[2,3-*d*]pyrimidines series against three fungal pathogens: *Candida albicans*, *Aspergillus fumigatus*, and *Rhizopus oryzae* [33]. Overall, all derivatives were more active on *A. fumigatus* and *R. oryzae* than on *C. albicans* (Table 32). Among all compounds evaluated, only three compounds (**73k–l** and **73n**) displayed moderate activity against *C. albicans*, 2-fold lower than the reference drug clotrimazole (MICs = 25 vs. 12.5 µg/mL). In contrast, compounds **73j** and **73m–o** were twice as active as clotrimazole (MICs = 50 vs. 100 µg/mL).

**Figure 9:**
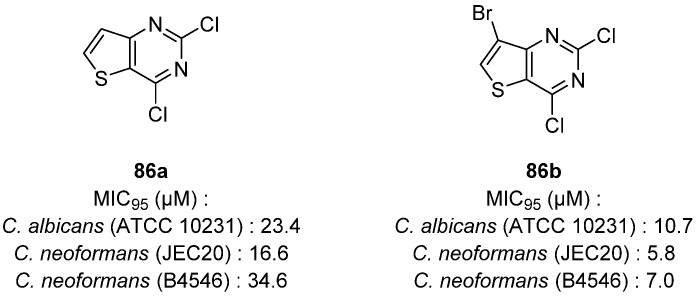
Structure and antifungal activity of halogenated thieno[3,2-*d*]pyrimidine derivatives **86**.

Temburnikar et al. reported the antifungal activity of two 2,4-dichlorothieno[3,2-*d*]pyrimidine derivatives against *Candida albicans* and *Cryptococcus neoformans* [25] (Figure 9). MIC_95_ values suggested that the introduction of a bromine atom in β position of the sulfur atom of the thiophene ring improved antifungal activity (**86b** vs. **86a**).

Thieno[2,3-*d*]pyrimidine derivatives **76a–d** reported by Tolba et al. were also highlighted as potent antifungal agents against *Geotrichum candidum*, *Candida albicans*, *Trichophyton rubrum*, and *Aspergillus flavus* [70]. Each compound displayed the same antifungal activity as clotrimazole against the four pathogens (MICs = 4.0 to 5.0 µg·mL^−1^, respectively, Table 33).

Thieno[2,3-*d*]pyrimidine derivatives **80a** and **80b**, reported having antibacterial activity (see previous section), were also evaluated for their potential antifungal activity against *Aspergillus fumigatus* (RCMB 002003), *Geotrichum candidum* (RCMB 052006), *Candida albicans* (RCMB 005002) and *Syncephalastrum racemosum* (RCMB 005003) [16]. Their antifungal efficacy was determined by their inhibition zone diameter compared to reference drugs (itraconazole and clotrimazole). Globally, **80a** and **80b** exhibited moderate activity on the four fungal strains (zone of inhibition = 10.6 to 15.1 mm, Table 34).

## 7. Thienopyrimidines with Antiviral Activity

### 7.1. Activity against Influenza A Virus

Influenza or flu is a recurrent respiratory infection in our modern society [77]. As the strains of influenza viruses vary from year to year, their circulation is closely monitored. Although influenza A infection is mostly mild, it can sometimes cause pneumonia and acute respiratory failure. Thus, in addition to the annual influenza vaccine, antiviral drugs remain essential to control influenza epidemics and pandemics. Looking for new anti-influenza molecules, Zhang et al. relied on the structure of pimodivir, an antiviral drug currently evaluated in phase III clinical trials [78]. This antiviral drug inhibits the trimeric RNA-dependent RNA polymerase of the virus. This enzyme is composed of three proteins, named polymerase basic protein 1 (PB1), polymerase basic protein 2 (PB2) and polymerase acidic protein (PA). The PB2 subunit generates 5′-capped RNA fragments from cellular pre-mRNA molecules used as primers for viral transcription [79]. The scaffold developed by Zhang et al. essentially conserved the 7-azaindole core of pimodivir and added a thienopyrimidine ring at position 3 (Table 35). Modifications such as the insertion of a thiophene ring, methylated (compounds **87c** and **87d**) or not (compounds **87a** and **87b**), were tolerated and the anti-influenza A activity remained in submicromolar EC_50_ values (Table 35). The most interesting compounds of this series, **87a** and **87b**, displayed an in vitro activity comparable to pimodivir (EC_50_ = 6.5 and 17 nM vs. EC_50_ = 4 nM for the reference drug). However, almost all compounds except **87c** presented quite high cytotoxicity on several cell lines (PBM, CEM, Vero, Huh7, and A549). Cytotoxicity of these compounds could be explained by the oxidation at position 2 of the 7-azaindole ring, engendered by aldehyde-oxidase. Therefore, a nitrogen atom was introduced in position 2 to prevent metabolization. This modification resulted in either maintenance of the antiviral activity (compounds **87e**) or a decrease in the antiviral activity (compound **87f**).

### 7.2. Activity against Hepatitis B Virus

Despite recent therapeutic advances, viral hepatitis still represents a major health issue worldwide [80]. Although a massive action of vaccination against hepatitis B virus (HBV) was carried out in endemic countries [81], the number of new cases remains substantial every year, associated with the development of active chronic forms and cirrhosis. Structurally, HBV is a double-stranded DNA virus and belongs to the hepadnavirus family.

In 2013, Al-Harbi and Abdel-Rahman proposed anti-HBV analogues of acyclovir, notably known for its anti-herpes virus properties [29], by replacing the guanine base by its thienopyrimidinone analogue. Preliminary SAR studies on thienopyrimidinone derivatives revealed that substitution at position 1 by a (2-hydroxyethoxy)methyl chain was essential to increase anti-HBV activity and to reach submicromolar EC_50_ values (Table 36). Molecule **90a** displayed the best activity of this series and similar activity to the antiviral reference drug lamivudine (EC_50_ = 0.2 and 0.1 µM, respectively). At position 7, several substituents on the cyclohexyl ring were tolerated, such as a methyl or a methoxy group (compounds **90a** to **90d**).

### 7.3. Activity against HIV-1

Human immunodeficiency virus (HIV) is a major contributor to the global burden of infectious diseases, with an estimated 38 million people living with this viral infection in 2019 [82]. The same year, the WHO counted 690,000 deaths due to HIV and 1.7 million new cases [83]. It mainly targets activated CD4 T lymphocytes and causes gradual depletion of this cell line, resulting in progressive immune dysfunction [84]. Eventually, progression of the disease reaching the immune deficiency syndrome (AIDS) state makes patients more vulnerable to opportunistic infections such as tuberculosis, pneumonia, cryptococcal meningitis or cytomegalovirus retinitis. Two types of viruses exist, HIV-1 and HIV-2, the former being predominant in humans. Antiretroviral therapy (ART) is the recommended treatment to decrease the viral load to concentrations below the limit of detection of available commercial assays. Unfortunately, resistance mutations to antiretroviral drugs tend to appear under selection pressure and may cause a loss of drug efficacy [85]. Therefore, the need to identify original bioactive compounds and new therapeutic targets remains a major concern.

#### 7.3.1. Thienopyrimidines as Reverse Transcriptase (RT) Inhibitors

A considerable work carried out by Kang et al. allowed the identification of new HIV-1 non-nucleoside reverse transcriptase inhibitors (NNRTIs) with EC_50_ in the nanomolar range [86]. The starting point of this work was the development of a thieno[3,2-*d*]pyrimidine scaffold derived from etravirine, a diarylpyrimidine (DAPY) NNRTI especially active against HIV-1. This series of compounds were evaluated for their anti-HIV activity and cytotoxicity in MT-4 cells infected by a wild-type (WT) HIV-1 strain (IIIB) and an HIV-2 strain (ROD). A lead compound **91a** was identified with an anti-HIV-1 activity 4-fold better than etravirine (Table 37) and low cytotoxicity (CC_50_ > 227 µM). Molecule **91a** showed good activity against mutant strains, with similar or better activity than etravirine (Table 38) and exhibited favorable in vivo pharmacokinetic parameters in rats. A SAR study demonstrated that the *para*-cyano group substituting the phenyl ring at position 4 played a key role in increasing antiviral activity (**91a** vs. **91b**). Furthermore, *para*-substitution of the *N*-benzylpiperidine group at R_2_ by several polar groups (unsubstituted sulfamide or amide) increased activity, whereas other groups with lower polarity (substituted sulfamide, fluorine, or ester) caused a drop in potency. Additional SARs based on compound **91a** revealed that a *para*-cyanovinyl substitution of the phenyl ring at position 4 increased non-nucleosidic reverse transcriptase inhibition (**91c**: EC_50_ (IIIB) = 1.22 nM) [87]. Compound **91c** exhibited better anti-HIV activity than both etravirine and compound **91a** against the K103N drug-resistant single mutant, and against F227L + V106A or K103N + Y181C (RES056) double mutants. Replacing the thieno[3,2-*d*]pyrimidine core by its thieno[2,3-*d*]pyrimidine isomer led to similar activity against WT HIV-1 strain and a slightly decreased activity on mutant strains compared to compound **91a**. Modification of the nitrogen atom position in the aminopiperidine group at position 2 (compound **91d**) led to a drop of activity (EC_50_s in the micromolar range) [87]. Similarly, diaminocyclohexane derivatives **91e** and **91f** showed a slight decrease in anti-HIV1 activity (EC_50_ = 7.1 and 10 nM, respectively) [88]. A diaminophenyl group at position 2 caused a loss of potency, with EC_50_s 10-fold weaker than etravirine (**91g** vs. ETV) [89]. Finally, various amino-cycloalkyl groups were introduced at position 2. Insertion of 8-azabicyclo[3.2.1]octane at position 2 (compound **91h**) was not tolerated whereas azepane (**91i**), pyrrolidine (**91j**) or azetidine (**91k**) derivatives showed moderate to high potency against HIV-1, with EC_50_ values between 2.20 and 217 nM on NL4-3 cell lines [90]. However, their activity was not evaluated on mutant strains.

**Figure 10:**
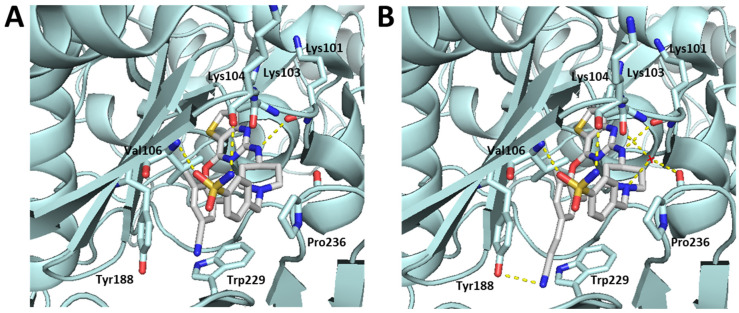
Co-crystallized structure of compounds **91a** (**A**) and **91b** (**B**) in the HIV-1 wild-type (WT) reverse transcriptase (RT) active site (PDB: 6C0J (**91a**) and 6C0N (**91b**)). The enzyme is represented as pale cyan cartoon mode and the compound is shown as stick representation. Key bonding interactions are indicated as yellow dotted lines.

In addition to these SAR studies, Yang et al. obtained co-crystal structures of **91a** and **91c** with HIV-1 wild-type (WT) reverse transcriptase (RT) and seven RT variants involved in drug-resistant mutations [91]. Ligand-enzyme interactions between HIV-1 WT reverse transcriptase and compounds **91a** or **91c** are presented in Figure 10. Several ligand–receptor interactions were common to both compounds: two hydrogen bonds between the primary sulfonamide group and Lys104 and Val106, one hydrogen bond of the NH-linker of the thienopyrimidine core and the piperidine ring with Lys101, and hydrophobic interactions with the phenoxy group and several aromatic amino-acids (e.g., Tyr188 and Trp219). In contrast, differences were observed, notably the hydrogen bond between the cyano group of **91b** and Tyr188, and the interaction of the nitrogen atom of the piperidine ring of **91b** with Lys103 and Pro236 via a hydrogen bond with a water molecule.

Further works carried out in the thieno[3,2-*d*]pyrimidine series allowed the identification of new NNRTI candidates. Sang et al. developed new thienopyrimidine derivatives based on biphenyl diarylpyrimidines (DAPYs) with nanomolar potency (EC_50_ = 7.8 to 526.2 nM) [28]. *Ortho*-substitution of the phenyl ring at position 4 was very well tolerated compared to *meta*-substitution that led to a drop of activity (EC_50_ = 7.8 nM for compound **92a** and 87.7 nM for compound **92b**, Table 39). *Ortho*-substitution was also in favor of high selectivity indexes compared to the reference drugs nevirapine and etravirine (SI = 28,346 vs. > 50 and > 833). Di-substitution was tolerated too. Compound **92a** displayed activity against HIV-1 similar to etravirine and 40-fold higher than nevirapine. Compounds **92c** and **92f** were almost 2-fold less active than di-fluorinated derivatives (compounds **92d** and **92e**), suggesting that only small substituents promote potency. All compounds presented lower cytotoxicity than the reference drugs nevirapine and etravirine (CC_50_ > 18.5 µM). Interestingly, all compounds demonstrated a better potency on HIV-1 mutant strains than nevirapine. However, none of these molecules presented a better activity against HIV-1 mutant strains than etravirine, except **92a**. Indeed, this latter compound showed similar activities on K103N and E138K, in the same range as etravirine (Table 40). Another SAR study on this series was reported thereafter [92]. The results demonstrated that only R_5_ and R_6_ substitutions were tolerated. An ortho-fluorinated substituent showed strong activity against HIV-1 IIIB strains in MT-4 cells (**7**: EC_50_ = 11 nM). Introduction of *ortho*-substituents such as a chlorine atom or a methyl group or introduction of *meta*-substituents such as a fluorine, a chlorine atom, or a methyl group led to moderate activity, better than or similar to nevirapine, but lower than etravirine. Then, combined substitutions at R_5_ and R_6_ were studied (Table 39). Four compounds, **92h** to **92k**, revealed a strong anti-HIV-1 activity (EC_50_ = 14 to 29 nM), similar or slightly weaker than etravirine. Concerning their activity on HIV mutant strains, **92h** to **92k** demonstrated lower potency than etravirine but a better activity than nevirapine.

Kankanala et al. focused their research on the catalytic domain of the HIV reverse transcriptase (RT) enzyme, named HIV RT-associated ribonuclease H (RNase H) [30]. Indeed, RT presents two distinctive active sites, an *N*-terminal DNA polymerase site and a *C*-terminal RNase H site. Four carboxylic residues interacting with two Mg^2+^ metal ions activate RNase. Therefore, a chelating group with the ability to complex two Mg^2+^ metal ions is essential to inhibit RNase. Inhibitors of RT RNase H described in the literature presented a metal chelate site and a peripheral hydrophobic group. Based on this model, Kankanala et al. synthesized a series of 3-hydroxythienopyrimidine-2,4-diones that were evaluated for their anti-RNase potential (Table 41). The first thiophene derivatives **93a** exhibited a potent activity with submicromolar IC_50_s and no polymerase (pol) inhibition. In contrast, a moderate integrase strand-transfer (INST) inhibition (IC_50_ = 4.5 μM), a low antiviral activity (EC_50_ = 11 μM) and an acceptable cytotoxicity on MAGI cells (CC_50_ = 28 μM) were observed. Regarding other derivatives, the *para*-halogen substitution of the phenyl ring at position 6 could cause a drop of either antiviral potency or cytotoxicity (compounds **93b** and **93c**). Regarding compound **93c**, *para*-chloro substitution led to a selectivity improvement, with an increase in RNase H inhibition (IC_50_ = 0.07 μM) and a decrease in INST inhibition. The main drawback of molecule **93c** was its low antiviral activity. To address this, bioisosteric analogues were synthesized and evaluated. Compound **94a** displayed similar activities to compound **93a** on RNase and INST. Replacing the phenyl group with a benzyl group at position 6 increased RNase inhibition (IC_50_ = 0.043 μM for compound **94b**). Substitution at position 5 by a phenyl ring, a hydrogen atom, or a methyl group led to submicromolar anti-RNase IC_50_ values (compounds **94c**, **94e–f**). In contrast, compound **94d** displayed the best activity in this series (IC_50_ = 0.040 μM). Substitution of both positions 5 and 6 maintained RNase H inhibition (compounds **94g–h**). To summarize, all compounds showed better RNase H inhibition and lower integrase strand-transfer activity than raltegravir, the reference drug chosen by the authors.

#### 7.3.2. Anti-HIV Thienopyrimidines with Other Mechanisms

Using a cell-based full-replication assay, Kim et al. identified 2-(phenylsulfonylmethyl)-thieno[3,2-*d*]pyrimidine derivatives able to inhibit the HIV-1 replication [20]. These compounds presented EC_50_ values in the micromolar or submicromolar range. The best compounds **95a** and **95i**, bearing a *para*-methoxypyridin-3-yl group at R_1_ (Table 42), presented a strong activity against HIV-1 virus replication (EC_50_ = 25 and 14 nM, respectively). Other *para*-substituents, such as halogen atoms (**95b**) or carbonyl group (**95c**) led to a loss of potency, highlighting that an electron-donating group at this position could be in favour of a good activity profile. Several derivatives bearing a heterocyclic group (pyridine (**95d**), morpholine (**95e**), thiophene (**95f**), or furan (**95g**)) or a substituted phenyl ring at R_1_ (**95h**) also showed lower activity on anti-HIV-1 replication. Other modifications at R_2_ were carried out by replacing the linker between the thienopyrimidine core and the 3-chlorophenyl group. Replacing the sulfone with a sulfide doubled the activity (**95i** vs. **95a**). Other modifications of the sulfone group (replacement by a carbon (**95j**), a carbonyl group (**95k**), etc.) led to a slightly to strongly decreased anti-HIV activity. Afterward, substituents on the phenyl ring at R_2_ were modulated. *Meta*-substitution was tolerated (**95i**), whereas di-substitution caused a loss of potency (compound **95l**). None of these compounds presented noticeable cytotoxicity on CEMx174-LTR-GFP CG8 cells and HeLa-LTR-GFP cells. The target involved in the HIV-1 replication inhibition remains to be identified.

**Figure 11:**
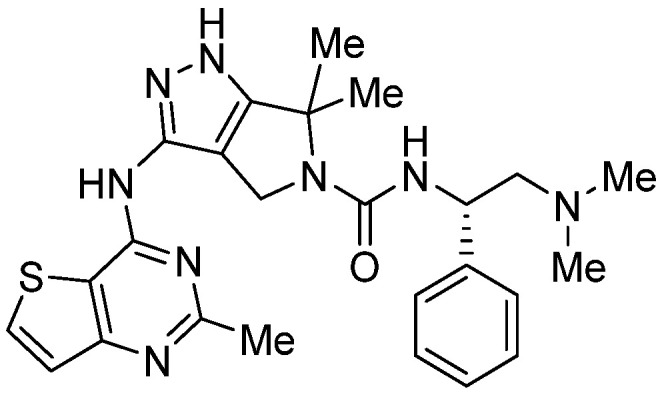
Structure of PF-3758309, an inhibitor of HIV-1 reversal latency.

During antiretroviral therapy, residual viraemia is present in the body. A quiescent form of the HIV-1 genome still replicates and persists in some CD4^+^ T cells [93]. Thus, research efforts focused on the eradication of these latently infected cells. In 2019, Vargas et al. demonstrated the inhibitory activity of various compounds on signaling pathways that blocked the reversal of HIV-1 latency [94]. Screening of compounds was carried out in the absence or presence of three mechanically distinct latency reversal agents (LRAs) called prostratin, panobinostat, and JQ-1. Compound PF-3758309 (Figure 11) which targets p-21-activated kinase 4 (PAK4), was identified as an inhibitor of LRAs. This means that it blocked the latency-reversing activity of prostratin, panobinostat, and JQ-1 (IC_50_ values equal to 0.07, 0.4, and 0.1 nM, respectively) in the HIV-1-latent 24ST1NLESG cell line. Furthermore, PF-3758309 revealed good selectivity. In addition, this molecule blocked cellular transcription of HIV-1 and virus reactivation in CD4^+^ T cells. Due to its mode of action, PF-3758309 could be associated to antiretroviral therapy to reduce the immune activation of CD4^+^ T caused by the presence of a lower rate of viraemia.

Overall, due to their structural relationship with purine bases, thienopyrimidines have been particularly studied as potential antiviral agents. The main works in this area have focused on the HIV virus and several compounds showed antiviral activity on HIV-1 or HIV-2 with micromolar or submicromolar values. Among the studied targets, several works focused on the reverse transcriptase of the virus, leading to the discovery of potent inhibitors, notably against various resistant strains.

## 8. Conclusions

After an in-depth study of the literature, thienopyrimidine emerges as an attractive scaffold in medicinal chemistry with a wide array of pharmacological properties. In this review, we have reported the distinct strategies currently used to access thienopyrimidine derivatives, as well essential information to design novel anti-infective agents and optimize their structures.

Among the different routes of synthesis studied, the construction of the pyrimidine ring from aminothiophene derivatives is the most used synthetic pathway. Introduction of various substituents on the pyrimidine and on the thiophene ring is quite easy, which allows access to a wide range of modulations. Moreover, SAR analysis reveals that thieno[3,4-*d*]pyrimidine derivatives are little studied as, to our knowledge, only one compound has been reported to have antibacterial and antifungal activities (compound **52**). In addition, antibacterial, antifungal and antitubercular agents are mostly thieno[2,3-*d*]thienopyrimidine derivatives, whereas compounds with antiviral activity are mainly represented by thieno[3,2-*d*]pyrimidines. Concerning antiparasitic agents, both thieno[2,3-*d*]pyrimidines or thieno[3,2-*d*]pyrimidines were reported. Finally, most of the compounds with anti-infective properties were identified after phenotypic screening and only few targets involved in their biological activity have been reported to date. When confirmed, enzymes have been identified as the main targets of these derivatives (protease, transferase, transcriptase, etc.).

## Figures and Tables

**Table 1:** The inhibitory activity of thienopyrimidine derivatives **54a–f** on FP-2.

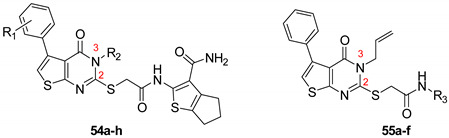					
Cpd	R_1_	R_2_	R_3_	Inhibitory Activity Against FP-2	
				Inhibition Rate at 10 μM (%)	IC_50_ (μM)
**54a**	H	Allyl	-	88.7	2.81
**54b**	H	Cyclohexyl	-	92.7	1.46
**54c**	H	3-F-Ph	-	79.0	2.05
**54d**	H	4-NO_2_-Ph	-	85.4	2.77
**54e**	H	4-Cl-Ph	-	84.7	4.30
**54f**	H	Benzyl	-	90.6	5.74
**54g**	4-Cl	Allyl	-	85.7	2.95
**54h**	3-NO_2_	Allyl	-	53.0	11.8
**55a**	-	-	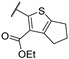	93.3	6.63
**55b**	-	-	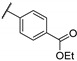	94.3	5.70
**55c**	-	-	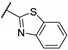	90.3	3.31
**55d**	-	-	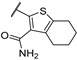	93.2	2.49
**55e**	-	-	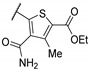	72.0	5.58
**55f**	-	-	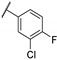	92.0	5.43

**Table 2:** Antiplasmodial activity of thieno[2,3-*d*]pyrimidines **56a–h**.

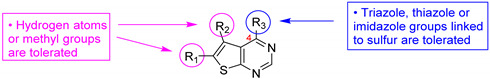						
Compound	R_1_	R_2_	R_3_	*P. falciparum*		Ref.
				NF54 EC_50_ (µM)	3D7 EC_50_ (µM)	
**56a**	H	H	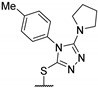	0.15	-	[48]
**56b**	H	H	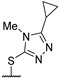	1.48	-	[48]
**56c**	H	H	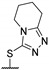	0.15	-	[48]
**56d**	Me	Me	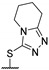	0.46	-	[48]
**56e**	H	H	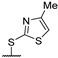	-	0.037	[49]
**56f**	H	H	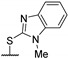	-	0.191	[49]
**56g**	H	H	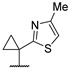	-	>23	[49]
**56h**	H	H	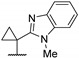	-	>23	[49]

**Table 3:** Biological evaluations of 4-thioether-thieno[2,3-*d*]pyrimidines **56i–o**.

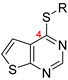						
Compound	R	% Inhibition, *Pf*NF54-Luc at 3 µM	*Pf*3D7 EC_50_ (µM)	*Pf*3D7 + 0.8 mM CoA EC_50_ (µM)	THP-1 EC_50_ (µM)	HEK-293 EC_50_ (µM)
**56i**	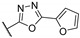	80.8 ± 0.5	0.283 ± 0.073	4.61 ± 0.15	6.13	>10 [48]
**56j**	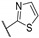	99.9 ± 0.0	0.0388 ± 0.0010	2.61 ± 0.42	27.5	>10
**56k**	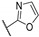	99.5 ± 0.5	0.0747 ± 0.021	2.58 ± 0.42	33.6	>10
**56l**	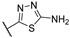	99.7 ± 0.1	0.0958 ± 0.0063	11.2 ± 2.6	11	>4
**56m**	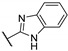	95.0 ± 1.1	0.0734 ± 0.0022	0.531 ± 0.007	16.5	>10
**56n**	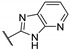	98.0 ± 0.2	0.149 ± 0.021	1.06 ± 0.03	28	>20
**56o**	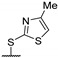	-	0.0370 ± 0.0017	1.51 ± 0.19	-	>40
**Chloroquine**	-	-	0.0195 ± 0.0034	0.0271 ± 0.0094	-	>40
**Artemisinin**	-	-	0.00337 ± 0.00065	0.00490 ± 0.0011	-	-

**Table 4:** SAR and antimalarial activity on *P. falciparum* resistant K1 strains and cytotoxicity on HepG2 cells of thieno[3,2-*d*]pyrimidin-4(3*H*)-one derivatives **57a–j**.

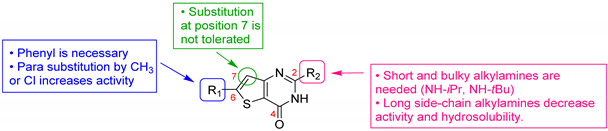				
Compound	R_1_	R_2_	Antiplasmodial Activity ^a^ on K1 EC_50_ (µM)	Cytotoxicity ^a^ on HepG2 CC_50_ (µM)
**57a**	4-Me-Ph	NH-*t*Bu	0.2 ± 0.02	25.6 ± 3.1
**57b**	4-Me-Ph	NH-*i*Pr	0.8 ± 0.5	49.4 ± 1
**57c**	4-Me-Ph	NH-*n*Pr	>5 ^b^	>62.5 ^b^
**57d**	Ph	NH-*t*Bu	1	12.5 ± 2.5
**57e**	H	NH-*t*Bu	>5 ^b^	8.4 ± 3.5
**57f**	3-Me-Ph	NH-*t*Bu	3.6	14.1 ± 1.4
**57g**	2-Me-Ph	NH-*t*Bu	1.7	4.0 ± 1.1
**57h**	4-Cl-Ph	NH-*t*Bu	0.8	15.0 ± 2.7
**57i**	4-F-Ph	NH-*t*Bu	>5 ^b^	5.1 ± 1.0
**57j**	4-Me-Ph	NH-*t*Bu·HCl	0.045	24.0
**Chloroquine ^c^**	-	-	0.5	30

^a^ The values are the means ± SD of three independent experiments. ^b^ No activity was observed at the highest concentration tested. ^c^ Antimalarial drug reference.

**Table 5:** Antimalarial activity of thieno[3,2-*d*]pyrimidine derivatives **58a–t**.

Compound	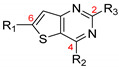			Activity on *P. falciparum* Strains EC_50_ (nM) ^a^		Degradation Half-Life (min)	Ref.
	R_1_	R_2_	R_3_	K1	NF54		
**58a**	Ph	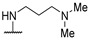	NHMe	73	28	344.9	[35]
**58b**	Br	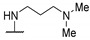	NHMe	973	636	-	[35]
**58c**	4-CN-Ph	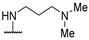	NHMe	19	9	238	[35]
**58d**	4-CF_3_-Ph	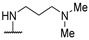	NHMe	32	29	124	[35]
**58e**	4-CF_3_O-Ph	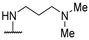	NHMe	26	28	>250	[35]
**58f**	3-CN-Ph	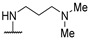	NHMe	17	4	-	[35]
**58g**	3-CF_3_-Ph	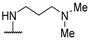	NHMe	-	13	31.1	[35]
**58h**	2-Cl-4-CF_3_-Ph	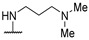	NHMe	47	16	130	[35]
**58i**	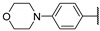	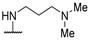	NHMe	12	7	15	[35]
**58j**	4-CF_3_-Ph	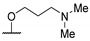	NHMe	>2436	1832	-	[54]
**58k**	4-CF_3_-Ph	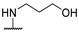	NHMe	233	111	-	[54]
**58l**	4-CF_3_-Ph	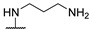	NHMe	>2608	>2608	-	[54]
**58m**	4-CF_3_-Ph	NH_2_	NHMe	-	1158	-	[54]
**58n**	4-CF_3_-Ph	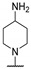	NHMe	-	42	-	[54]
**58o**	4-CF_3_-Ph	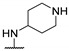	NHMe	24	19	-	[54]
**58p**	4-CF_3_-Ph	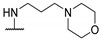	NHMe	-	43	23.9	[54]
**58q**	4-CF_3_-Ph	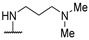	NH-benzyl	-	42	-	[54]
**58r**	4-CF_3_-Ph	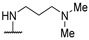	NH_2_	-	58	104	[54]
**58s**	4-CF_3_-Ph	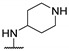	NH_2_	24	25	>150	[54]
**58t**	4-CF_3_-Ph	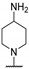	NH_2_	33	20	>150	[54]
**Chloroquine ^b^**				194	16	-	[54]
**Artesunate ^b^**				3	4	-	[54]

^a^ Mean from n values of ≥2 independent experiments with multi-drug-resistant (K1) and sensitive (NF54) strains of *P. falciparum*. ^b^ Antimalarial drug references.

**Table 6:** SAR studies and inhibition profile of thienopyrimidine derivatives against *P. falciparum* D6.

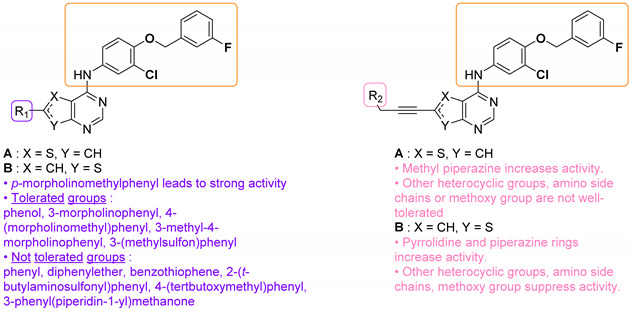					
Compound		R_1_	R_2_	*P. falciparum* D6 EC_50_ (µM) (r^2^) ^a^	Ref.
**59a**	A	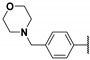	-	0.027 (0.97)	[14]
**59b**	B			0.089 (0.81)	
**59c**	A	-	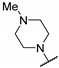	0.64 (0.99)	[57]
**59d**	B			0.26 (0.94)	

^a^ Compounds screened against *P. falciparum* (D6 strain) either in duplicate or quadruplicate and had r^2^ values >0.90 except for **59b** (r^2^ = 0.81).

**Table 7:** SAR data from Desroches et al.’s studies.

R		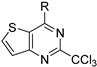	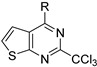
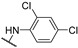	K1 *P. falciparum* IC_50_ (µM)	0.6	0.5
	HepG2 CC_50_ (µM)	3.2	6.2
	SI^a^	5.3	12.4
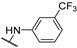	K1 *P. falciparum* IC_50_ (µM)	0.9	0.6
	HepG2 CC_50_ (µM)	0.7	6.2
	SI ^a^	0.8	6.7
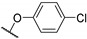	K1 *P. falciparum* IC_50_ (µM)	0.6	-
	HepG2 CC_50_ (µM)	4.3	-
	SI ^a^	7.2	-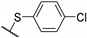	K1 *P. falciparum* IC_50_ (µM)	0.4	0.8
	HepG2 CC_50_ (µM)	6.9	6.2
	SI ^a^	17.2	16.6
**Doxycycline ^b^**	K1 *P. falciparum* IC_50_ (µM)	6.0	
	HepG2 CC_50_ (µM)	20	
	SI ^a^	3.3	
**Atovaquone ^b^**	K1 *P. falciparum* IC_50_ (µM)	0.001	
	HepG2 CC_50_ (µM)	>15.6	
	SI^a^	15,600	
**Chloroquine ^b^**	K1 *P. falciparum* IC_50_ (µM)	0.6	
	HepG2 CC_50_ (µM)	30	
	SI ^a^	50	
**Doxorubicine**	HepG2 CC_50_ (µM)	0.2	

^a^ SI: Selectivity Index as a ratio of Hep G2 CC_50_/K1 EC_50_. ^b^ Doxycycline, atovaquone, and chloroquine were used as antimalarial reference drugs.

**Table 8:** Antitrypanosomatid activity of thienopyrimidine derivatives **59a–g** and **60a–b**.

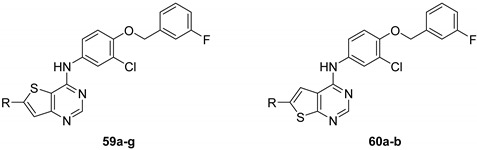					
Cpd	R	*L. major* Amastigotes EC_50_ (µM) (r^2^) ^a^	*L. major* Promastigotes EC_50_ (µM) (r^2^) ^a^	*T. brucei brucei* EC_50_ ± SEM (µM)	*T. cruzi* Amastigotes EC_50_ ± SEM (µM)
**59a** **(GW837016X)**	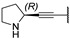	-	-	0.26	-
**59b**	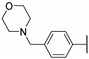	>15	0.22 (0.84)	1.1 ± 0.0	>50.0
**59c**	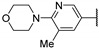	>3	>3	0.084 ± 0.0	3.3 ± 1.2
**59d**	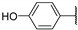	1.58 (0.83)	>20	2.2 ± 0.1	0.75 ± 0.02
**59e**	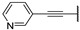	0.38 (0.94)	-	1.3 ± 0.3	12 ± 0.45
**59f**	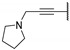	4.1 (0.77)	-	0.28 ± 0.07	1.8 ± 0.17
**59g**	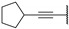	>15	-	0.76 ± 0.07	>50
**60a**	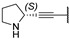	4.2 (0.94)	-	0.22 ± 0.02	3.7 ± 0.23
**60b**	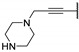	9.1 (0.88)	-	1.9 ± 0	0.61 ± 0.16
**Amphotericin B ^b^**	-	0.035 (0.90)	-	-	-
**Suramin ^b^**	-	-	-	0.04 ± 0	-
**Benznidazole ^b^**	-	-	-	-	0.79 ± 0.01

^a^ Compounds screened against *L. major* amastigotes and promastigotes were tested in duplicate and had r^2^ values >0.75. ^b^ Drug references.

**Table 9:** Thienopyrimidine derivatives as selective inhibitors of *L. donovani* and *L. major* NMT.

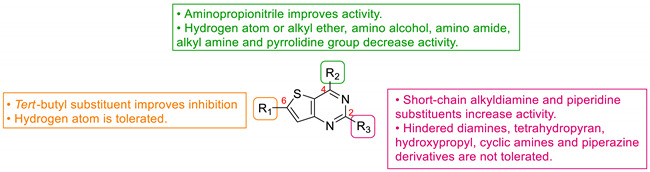						
Cpd	R_1_	R_2_	R_3_	CMP Assay IC_50_ (μM)		
				*Ld*NMT	*Lm*NMT	*Hs*NMT1
**61a**	H	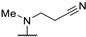	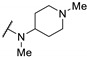	0.34	0.20	5.7
**61b**	H		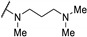	0.15	2.7	>100
**61c**	H		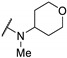	>100	-	-
**61d**	H		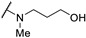	>100	-	-
**61e**	H		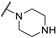	28	-	-
**61f**	H		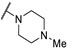	30	-	-
**61g**	H		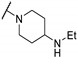	7.7	-	46
**61h**	H		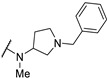	>100	-	-
**61i**	H		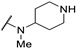	0.5	-	11
**61j**	H		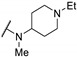	0.83	0.62	17
**61k**	H		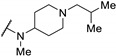	4.0	-	>100
**61l**	H		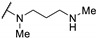	5.2	-	-
**61m**	H		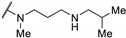	>100	-	-
**61n**	H		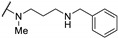	>100	-	-
**61o**	H	H	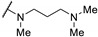	>100	-	-
**61p**	H	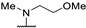		44	-	-
**61q**	H	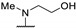		22	36	-
**61r**	H	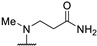		-	13	-
**61s**	H	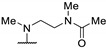		>100	-	>100
**61t**	H	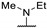		>100	-	-
**61u**	H	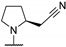		3.6	-	16
**61v**	H		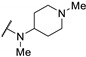	0.15	-	>100
**61w**	*t*-Bu	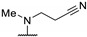	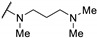	0.36	0.16	-
**61x**	*t*-Bu		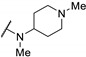	0.046	0.042	0.55

**Table 10:** Antihelminthic activity of thienopyrimidine derivatives against *Trichinella spiralis*.

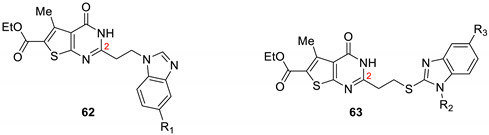					
Compound	R_1_	R_2_	R_3_	Efficacy (%) ^a^ after 24 h ^b^ 5 μg/mL	Efficacy (%) after 48 h 5 μg/mL
**62a**	H	-	-	79.8	85.30
**63a**	-	Et	H	39.07	59.75
**63b**	-	H	NO_2_	50.00	80.05
**63c**	-	H	Cl	0.00	5.09
**Albendazole** **(20 μg/mL)**	-	-	-	10.8	14.8

^a^ Control—96 parasites. ^b^ *p* > 0.05.

**Table 11:** Antituberculosis activity of thieno[2,3-*d*]pyrimidine derivatives **64–64g**.

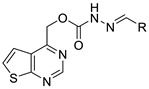			
Compound	R	*M. tuberculosis* H37Rv MIC (µM)	THP-1 IC_50_ (µM)
**64a**	Ph	320	ND ^a^
**64b**	4-((Me)_2_)N-Ph	70	2492.90
**64c**	2-Br-Ph	64	2387.80
**64d**	2-NO_2_-Ph	70	2415.90
**64e**	4-Cl-Ph	71	2264.80
**64f**	4-MeO-Ph	66	2454.70
**64g**	3,4,5-(MeO)_3_-Ph	62	2019.90
**Pyrazinamide**	-	60.97	ND

^a^ ND: not determined.

**Table 12:** Thienopyrimidin-4-one derivatives with antimycobacterial activity.

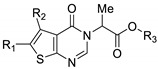				
Compound	R_1_	R_2_	R_3_	TB IC_90_ (µg·mL^−1^)
**65a**	H	4-Me-Ph	CH_2_Ph-Cl-4	1.0
**65b**	H	3,4-(Me)_2_-Ph	Cyclohexyl	1.7
**65c**	CH_3_	Me	Cyclohexyl	1.8
**65d**	H	4-Br-Ph	CH_2_Ph	2.2
**65e**	H	4-Et-Ph	Et	6.6

**Table 13:** 4-amino-substituted thienopyrimidine derivatives with antimycobacterial activity.

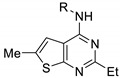		
Compound	R	MABA in *M. tuberculosis* IC_50_ (µM)
**66a**	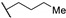	2.7
**66b**	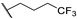	0.11
**66c**	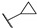	0.62
**66d**	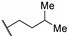	0.32
**66e**	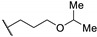	0.32
**66f**	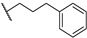	0.083
**66g**	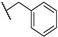	0.15
**Bedaquiline**	-	<0.078
**Thioridazine**	-	11.2

**Table 14:** Thieno[2,3-*d*]pyrimidine derivatives as potent PglD inhibitors.

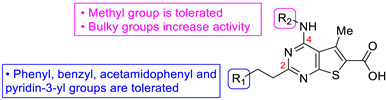			
Compound	R_1_	R_2_	*C. jejuni* PglD IC_50_ (µM)
**67a**	Ph	Me	2.2 ± 0.4
**67b**	Ph	PhCH_2_CH_2_	1.4
**67c**	Ph	4-MeO-PhCH_2_CH_2_	0.54
**67d**	Ph	4-F-PhCH_2_CH_2_	0.42
**67e**	Ph	4-Me-PhCH_2_CH_2_	0.72
**67f**	Ph	3,5-(MeO)_2_-PhCH_2_CH_2_	0.37
**67g**	Ph	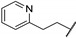	0.42
**67h**	Ph	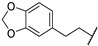	0.59
**67i**	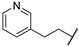	Me	0.46 ± 0.05
**67j**	4-AcNH-Ph	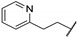	0.28
**67k**	4-AcNH-Ph	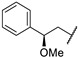	0.27 ± 0.09
**67l**	Benzyl	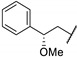	0.87
**67m**	Benzyl	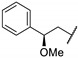	0.42

**Table 15:** Antibacterial activity of thieno[2,3-*d*]pyrimidin-4- (or 2,4-di)ones derivatives.

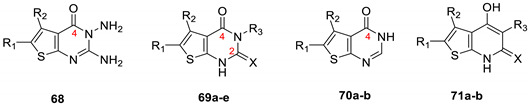									
Compound	R_1_	R_2_	R_3_	X	Inhibition Zone Diameter (mm)				Ref.
					*S. aureus*	*E. coli*	*B. cereus*	*P. aeruginosa*	
**68**	COPh	Me	-	-	26	24	27	NT ^a^	[31]
**69a**	COPh	Me	Ph	S	24	23	26	NT	[31]
**69b**	COPh	Me	Ph	O	22	21	23	NT	[31]
**69c**	COPh	Me	H	S	21	20	22	NT	[31]
**71a**	COPh	Me	COMe	O	9	10	12	NT	[31]
**71b**	COPh	Me	CN	O	11	12	14	NT	[31]
**70a**	Me	Me	-	-	6	6	6	6	[11]
**70b**	Me	NO_2_	-	-	12	15	16	10	[11]
**69d**	Me	Me	H	O	8	16	8	6	[11]
**69e**	Me	Me	H	S	12	6	12	8	[11]
**Levofloxacin**	-	-	-	-	26	30	28	NT	[31]

^a^ NT: not tested.

**Table 16:** Antibacterial activity of spiro thieno[2,3-*d*]pyrimidine-4-one derivatives **72a–h**.

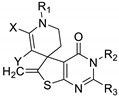									
Cpd	X	Y	R_1_	R_2_	R_3_	Antibacterial Activity MIC (µM)			
						*S. agalactiae* (1) ^a^	*S. agalactiae* (2) ^a^	*E. faecalis* ^a^	*S. epidermidis* ^a^
**72a**	H	CO_2_Me	Bn	H	CH_2_OMe	25	25	25	>250
**72b**	H	CO_2_Me	*i*Pr	H	CH_2_Cl	25	25	25	>250
**72c**	H	CO_2_Me	Et	Ph	H	25	25	25	>250
**72d**	H	CO_2_Me	Et	4-Cl-Ph	H	125	25	25	25
**72e**	H	COMe	Et	4-Cl-Ph	H	>250	250	250	250
**72f**	CO_2_Me	CO_2_Me	Et	4-Cl-Ph	H	>250	250	NT ^b^	>250
**72g**	H	CO_2_Me	Et	4-NO_2_-Ph	H	250	250	NT	>250
**72h**	H	CO_2_Me	Et	4-MeO-Ph	H	25	25	25	250
**Ampicillin**	-	-	-	-	-	1.5	1.5	25	25

^a^ Resistance profiles were evaluated by antimicrobial susceptibility testing. *S. agalactiae* (1): tetracycline; *S. agalactiae* (2): clindamycin, erythromycin, tetracycline; *E. faecalis*: chloramphenicol, rifampicin, Synercid; *S. epidermidis*: fosfomycin, ampicillin, and penicillin G (β-lactamase positive). ^b^ NT: not tested.

**Table 17:** Cytotoxic activity of spiro thieno[2,3-*d*]pyrimidine-4-one derivatives **72b–d**.

Compound	Cancer Cell Lines ^a^			
	GI_50_ ^b^ (µM)			
	MDA-MB-231	OV2008	HepG2	C6
**72b**	8	7	21	23
**72d**	44	16	40	48

^a^ MDA-MB-231: human breast carcinoma cells, OV2008: human ovarian carcinoma cells, HepG2: human liver carcinoma cells, C6: rat glioma cell line. ^b^ GI_50_: concentration of half-maximal inhibition of cell proliferation.

**Table 18:** Antibacterial activity of thieno[2,3-*d*]pyrimidin-4-one derivatives **73a–i**.

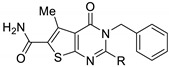				
Compound	R	MIC (µg/mL)		
		Gram Positive	Gram Negative	
		*B. subtitlis*	*P. aeruginosa*	*P. vulgaris*
**73a**	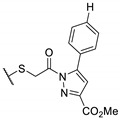	50	50	25
**73b**	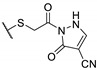	25	25	100
**73c**	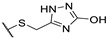	50	25	100
**73d**	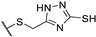	100	100	25
**73e**	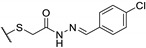	25	25	100
**73f**	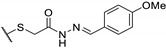	100	100	25
**73g**	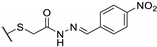	25	25	100
**73h**	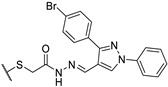	25	25	100
**73i**	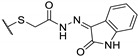	100	100	25
**Ampicillin**	-	12.5	125	-
**Levofloxacin**	-	-	12.5	12.5

**Table 19:** Antibacterial activity of 5-furyl thieno[2,3-*d*]pyrimidin-4-ones derivatives.

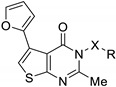						
Compound	X	R_2_	Antibacterial Activity MIC (µmol·L^−1^)			
			Gram-Positive Bacteria		Gram-Negative Bacteria	
			*S. aureus*	*B. subtilis*	*E. coli*	*S. typhi*
**74a**	NHCO	4-F-Ph	9	9	9	11
**74b**	NHCO	4-MeO-Ph	10	9	10	12
**74c**	NHCO	4-NO_2_-Ph	6	5	7	6
**74d**	NHCO	2,4-F_2_-Ph	4	5	4	5
**74e**	NCH	4-F-Ph	10	9	9	8
**74f**	NCH	4-MeO-Ph	11	11	12	12
**74g**	NCH	4-NO_2_-Ph	4	5	7	7
**74h**	NCH	2,4-F_2_-Ph	5	4	5	6
**Ampicillin**	-	-	50	4	4	49
**Penicillin-G**	-	-	6	22	5	5
**Chloramphenicol**	-	-	4	6	5	6

**Table 20:** Antibacterial activity of thieno[2,3-*d*]pyrimidine derivatives **76a–d**.

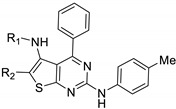						
Compound	R_1_	R_2_	Antibacterial Activity MIC (µg·mL^−1^)			
			Gram Positive		Gram Negative	
			*B. cereus*	*S. aureus*	*P. aeruginosa*	*E. coli*
**76a**	H	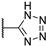	5.0	5.0	4.0	5.0
**76b**	H	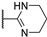	4.0	5.0	5.0	4.0
**76c**	COCH_2_Cl	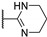	4.0	5.0	4.0	5.0
**76d**	COCH_2_NHPh	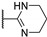	5.0	4.0	5.0	8.0
**Reference drugs**	-	-	5.0 Ofloxacin	4.0 Levofloxacin	4.0 Clindamycin	5.0 Nitrofurantoin

**Table 21:** Antibacterial activity of thieno[2,3-*d*]pyrimidine derivatives **77a–i**.

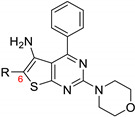					
Compound	R	Zone of Bacterial Inhibition at 10 mg/mL (in mm)			
		Gram Positive		Gram Negative	
		*S. aureus*	*B. subtilis*	*Salmonella* sp.	*E. coli*
**77a**	CN	0	14	14	0
**77b**	CONH_2_	0	16	0	0
**77c**	CO_2_Et	0	0	14	0
**77d**	COPh	0	15	0	0
**77e**	COMe	0	15	14	0
**77f**	CONHPh	0	15	0	0
**77g**	CONHPh-Cl-4	15	0	0	0
**77h**	CONHPh-OMe-4	0	15	0	0
**77i**	CONHPh-Br-4	0	0	0	20
**Ampicillin**	-	23	32	-	-
**Gentamycin**	-	-	-	17	19

**Table 22:** Antibacterial activity of 2-furyl thieno[2,3-*d*]pyrimidine derivatives **78a–g**.

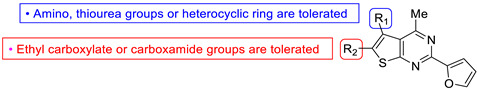						
Compound	R_1_	R_2_	Inhibition Zone Diameter (mm/mg Sample)			
			Gram Positive		Gram Negative	
			*B. subtilis*	*S. aureus*	*E. coli*	*P. aeruginosa*
**78a**	NH_2_	CO_2_Et	17	19	14	10
**78b**	NH_2_	CONHNH_2_	18	18	10	14
**78c**	NH_2_	CONHPh	14	18	16	10
**78d**	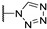	CO_2_Et	15	13	16	15
**78e**	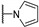	CO_2_Et	11	15	10	17
**78f**	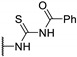	CO_2_Et	13	17	19	19
**78g**	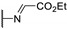	CO_2_Et	20	16	22	21
**Ampicillin**	-	-	26	21	25	26

**Table 23:** Antibacterial activity of 4-aminophenyl- or 4-pyrazolyl thieno[2,3-*d*]pyrimidine derivatives.

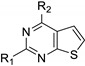								
Cpd	R_1_	R_2_	Inhibition Zone (mm)					Ref.
			*E. coli*	*B. sphaericus*	*B. subtilis*	*S. aureus*	*K. pneumonia*	
**79a**	Cl	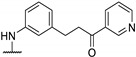	16 **^a^**	8 **^a^**	-	-	-	[72]
**79b**	Cl	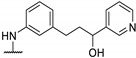	17 **^a^**	10 **^a^**	-	-	-	[72]
**79c**	Cl	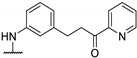	11 **^a^**	0 **^a^**	-	-	-	[72]
**79d**	4-CF_3_-Ph	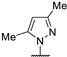	15.5 ^b^	-	11.5 ^b^	12.5 ^b^	14.5 ^b^	[27]
**79e**	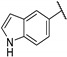	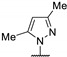	16.5 ^b^	-	12.5 ^b^	14.5 ^b^	15 ^b^	[27]
**79f**	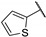	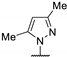	17 ^b^	-	13 ^b^	15 ^b^	16.5 ^b^	[27]
**79g**	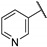	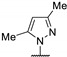	13 ^b^	-	11 ^b^	11.5 ^b^	12.5 ^b^	[27]
**Amoxicillin**	-	-	19.6 ^b^	-	15.7 ^b^	17.4 ^b^	18 ^b^	[27]

^a^ Experiments were realized at a concentration of 80 µL/mL. ^b^ Experiments were realized at a concentration of 100 µL/mL.

**Table 24:** Kanawade et al. derivatives as antibacterial agents.

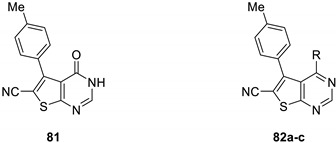					
Compound	R	Antibacterial Activity MIC (µg/mL)			
		Gram-Negative Bacteria		Gram-Positive Bacteria	
		*E. coli*	*P. aeruginosa*	*S. aureus*	*S. pyogenes*
**81**	-	100	62.5	200	250
**82a**	Piperazinyl	125	250	62.5	100
**82b**	Piperazinyl-carboxylate	62.5	100	250	250
**82c**	4-Cl-Ph	125	62.5	200	100
**Ampicillin**	-	100	NA ^a^	250	100
**Chloramphenicol**	-	50	50	50	50
**Ciprofloxacin**	-	25	25	50	50

^a^ NA: not active.

**Table 25:** Thieno[3,2-*d*]pyrimidine derivatives incorporating an acylhydrazone motif as antibacterial agents.

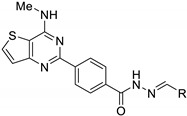					
Compound	R	Zone of Inhibition of the Bacteria (mm, C = 30 µL)			
		*E. coli*	*Pseudomonas* sp.	*S. aureus*	*Bacillus* sp.
**83a**	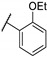	13	17	25	17
**83b**	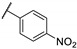	14	5	14	NA ^a^
**83c**	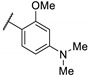	NA ^a^	NA ^a^	17	NA ^a^
**83d**	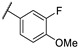	15	14	16	15
**83e**	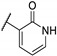	15	14	19	NA ^a^
**83f**	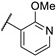	NA ^a^	14	NA ^a^	NA ^a^
**Streptomycin**	-	35	32	34	37

^a^ NA: not active.

**Table 26:** Antibacterial activity of thieno[3,2-*d*]pyrimidin-4-one derivatives **84a–j**.

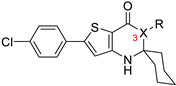								
Cpd	X	R	MIC (µmol·L^−1^)					
			Gram-Negative Bacteria			Gram-Positive Bacteria		
			*E. coli*	*K. pneumoniae*	*P. aeruginosa*	*S. lactis*	*S. aureus*	*E. faecalis*
**84a**	O	-	8	8	7	6	5	8
**84b**	N	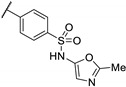	2	3	2	2	2	4
**84c**	N	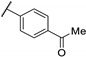	7	7	5	5	4	7
**84d**	N	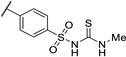	6	5	5	5	4	6
**84e**	N	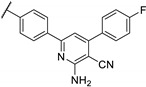	4	3	3	2	2	4
**84f**	N	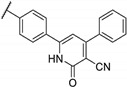	9	7	6	5	5	7
**84g**	N	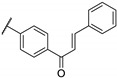	7	8	7	8	6	8
**84h**	N	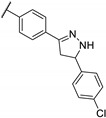	3	2	3	2	1	2
**84i**	N	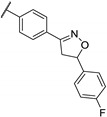	3	3	2	3	2	4
**84j**	N	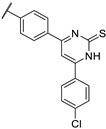	2	1	4	1	2	3
**Ciprofloxacin**	-	-	5	4	4	2	2	4

**Table 27:** New 7-nitro-thieno[3,2-*d*]pyrimidine derivatives as anti *C. difficile* agents.

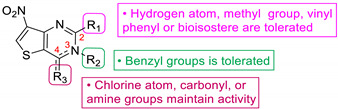					
Compound	R_1_	R_2_	R_3_	*C. difficile* MIC (µg·mL^−1^)	
				ATCC BAA 1870	ATCC 43255
**85a**	H	H	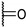	4	2
**85b**	Me	H	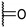	4	8
**85c**	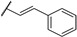	H	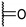	4	16
**85d**	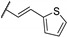	H	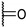	4	4
**85e**	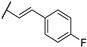	H	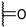	1	2
**85f**	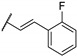	H	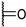	2	4
**85g**	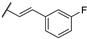	H	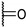	4	4
**85h**	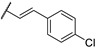	H	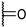	2	2
**85i**	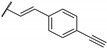	H	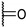	2	4
**85j**	Me	Benzyl	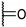	2	16
**85k**	Me	4-NO_2_-PhCH_2_	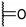	2	4
**85l**	Me	H	Cl	4	8
**Vancomycin**	-	-	-	1	0.5
**Metronidazole**	-	-	-	0.125	0.25
**Fidaxomicin**	-	-	-	0.0625	0.0625

**Table 28:** Antibacterial activity of thienopyrimidines **52** and **53**.

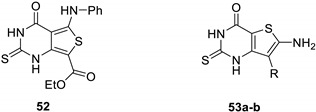			
Compounds	R	Inhibition Zone Diameter (mm)	
		*P. aeruginosa*	*S. aureus*
**52**	-	13	12
**53a**	CN	14	14
**53b**	CO_2_Et	13	14
**Tetracyclin**	-	28	26

**Table 29:** Antifungal activity of thienopyrimidines **68–69** and their thienopyridine analogues **71**.

Compound	Inhibition Zone Diameter (mm) ^a^	
	*C. albicans*	*A. niger*
**68**	26	30
**69a**	24	28
**69b**	22	26
**69d**	23	25
**71a**	11	15
**71b**	13	17
**Nystatin**	27	26

^a^ Inhibition diameter was measured after 24 h of incubation.

**Table 30:** Antifungal activity of 2-thioxothienopyrimidin-4(3*H*)-ones **52** and **53a–b**.

Compound	Inhibition Zone Diameter (mm/g of Samples)	
	*A. flavus*	*C. albicans*
**52**	0	10
**53a**	13	14
**53b**	14	13
**Amphotericin B**	16	19

**Table 31:** Antifungal activity of thieno[3,2-*d*]pyrimidin-4-ones **84k–q**.

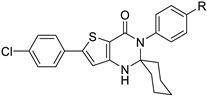				
Compound	R	MIC (µmol·mL^−1^)		
		*C. albicans*	*A. flavus*	*G. lucidum*
**84k**	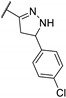	4	5	4
**84l**	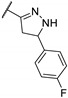	2	2	1
**84m**	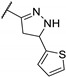	3	2	2
**84n**	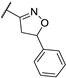	7	4	6
**84o**	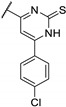	5	4	4
**84p**	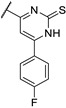	4	3	3
**84q**	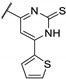	4	4	5
**Ketoconazole**	-	3	2	3

**Table 32:** Antifungal activity of thieno[3,2-*d*]pyrimidin-4-ones **73j–p**.

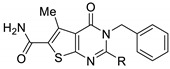				
Compound	R	MIC (µg/mL)		
		*C. albicans*	*A. fumigatus*	*R. oryzae*
**73j**	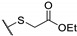	100	50	50
**73k**	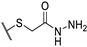	25	100	50
**73l**	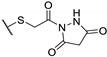	25	50	100
**73m**	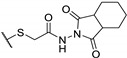	100	50	50
**73n**	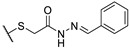	25	50	50
**73o**	OEt	50	50	50
**73p**	OH	100	100	50
**Clotrimazole**	-	12.5	100	100

**Table 33:** Antifungal activity of thieno[2,3-*d*]pyrimidines **76a–b**.

Compound	Antifungal Activity MIC (µg·mL^−1^)			
	*G. candidum*	*C. albicans*	*T. rubrum*	*A. flavus*
**76a**	4.0	5.0	4.0	5.0
**76b**	4.0	5.0	4.0	5.0
**76c**	4.0	5.0	4.0	5.0
**76d**	4.0	-	4.0	5.0
**Clotrimazole**	4.0	5.0	4.0	5.0

**Table 34:** Antifungal activity of compounds **80a–b**.

Compound	Inhibition Zone Diameter ^a^ (mm)			
	*A. fumigatus*	*G. candidum*	*C. albicans*	*S. racemosum*
**80a**	12.5 ± 0.09	12.8 ± 0.1	11.0 ± 0.05	13.4 ± 0.08
**80b**	15.1 ± 0.01	14.4 ± 0.1	13.4 ± 0.4	10.6 ± 0.2
**Itraconazole**	28 ± 0.05	27 ± 0.1	26 ± 0.02	22 ± 0.09
**Clotrimazole**	26 ± 0.1	23 ± 0.03	18 ± 0.1	20 ± 0.2

^a^ Concentration at 10 mg·mL^−1^.

**Table 35:** RSA, anti-influenza A activity, and cytotoxicity of thienopyrimidine derivatives **87a–f**.

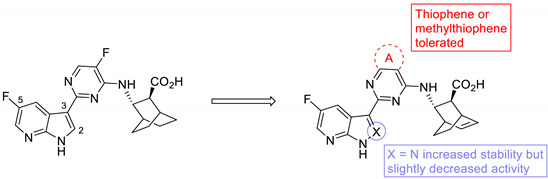								
Compound	Structure		Anti-Influenza A Activity in A549 Cells EC_50_ (nM ± SD)	Cytotoxicity CC_50_ (µM)				
	X	A		PBM	CEM	Vero	Huh7	A549
**87a**	C	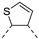	6.5 ± 1.1	9.3	4.2	65.9	24.0	14.5
**87b**	C	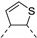	17 ± 10	64.6	31.6	74.9	68.7	14.7
**87c**	C	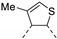	29 ± 6	>100	>100	>100	>100	>100
**87d**	C	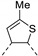	27 ± 14	78.4	12.9	90.3	31.2	41.7
**87e**	N	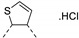	12 ± 1	>100	13.7	>100	62.7	>100
**87f**	N	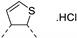	42 ± 7	>100	67.0	52.5	70.7	32.5
**Pimodivir**	-	-	4 ± 2	>100	48.9	>100	95.8	20.0

**Table 36:** Thieno[3,2-*d*]pyrimidine activity against HBV DNA production.

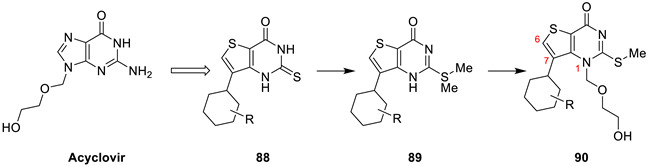		
Compound	R	HBV DNA EC_50_ (µM)
**88**	4-Me	1.7
**89**	4-Me	1.6
**90a**	4-Me	0.2
**90b**	H	0.7
**90c**	2-Me	0.6
**90d**	4-MeO	0.6
**Lamivudine ^a^**	-	0.1

^a^ Drug reference.

**Table 37:** Anti-HIV activity of thieno[3,2-*d*]pyrimidine derivatives **91a–l**.

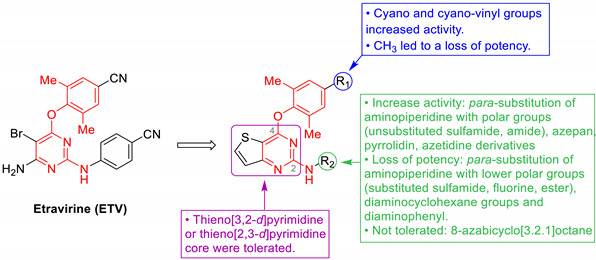						
Cpd	R_1_	R_2_	EC_50_^a^ (nM)			Ref.
			IIIB	ROD	NL4-3	
**91a**	CN	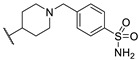	1.4 ± 0.4	>227,890	1.16 ± 0.43	[86,90]
**91b**	Me	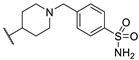	3552 ± 848	>16,460	-	[86]
**91c**	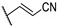	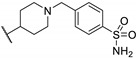	1.22 ± 0.26	>2.30	-	[87]
**91d**	CN	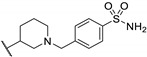	>1.17 × 10^3^	>1.17	-	[87]
**91e**	CN	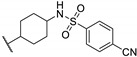	7.1 ± 0.5	>9.287	-	[88]
**91f**	CN	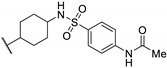	10 ± 8	>3734	-	[88]
**91g**	CN	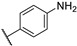	58 ± 29	>19,390	-	[89]
**91h**	CN	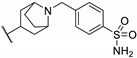	-	-	>217	[90]
**91i**	CN	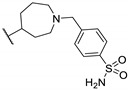	-	-	2.20 ± 0.67	[90]
**91j**	CN	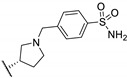	-	-	8.69 ± 2.74	[90]
**91k**	CN	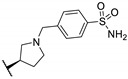	-	-	104 ± 36.8	[90]
**91l**	CN	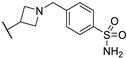	-	-	4.53 ± 1.30	[90]
**ETV**	-	-	5.8 ± 4.0	-	-	[86]

^a^ EC_50_: Concentration of compounds required to achieve 50% protection of TZM-bl cell lines (NL4-3) and MT-4 cell cultures (IIIB and ROD) against HIV-1-induced cytotoxicity, presented as the mean ± standard deviation (SD) and determined by the MTT method.

**Table 38:** Anti-HIV-1 activity of thieno[3,2-*d*]pyrimidine derivatives against HIV-1 mutant strains.

Cpd	EC_50_^a^ (nM)							Ref.
	L100I	K103N	Y181C	Y188L	E138K	F227L + V106A	RES056 (K103N + Y181C)	
**91a**	3.4 ± 0.6	2.9	3.2 ± 0.4	3.0 ± 0.1	2.9	4.2 ± 1.2	30.6 ± 12	[86]
**91b**	4519 ± 158	4937 ± 407	4845 ± 118	8471 ± 197	5505 ± 315	4547 ± 13	>16,462	[86]
**91c**	1.34 ± 0.5	0.958 ± 0.07	5.00 ± 0.1	5.45 ± 0.2	4.74 ± 0.2	2.70 ± 1.74	5.50 ± 0.811	[87]
**91d**	>1.17 × 10^3^	>1.17 × 10^3^	>1.17 × 10^3^	>1.17 × 10^3^	>1.17 × 10^3^	>1.17 × 10^3^	>1.17 × 10^3^	[87]
**91e**	424 ± 361	70 ± 25	428 ± 294	675 ± 91	45 ± 1	3583 ± 241	>9280	[88]
**91f**	562 ± 487	32 ± 2	513 ± 415	903 ± 248	35 ± 1	1208 ± 333	>3727	[88]
**91g**	280 ± 61	14 ± 1	780 ± 9	790 ± 43	31 ± 4	770 ± 110	>19,390	[89]
**ETV**	5.4 ± 2.1	2.4 ± 0.6	15.8 ± 2.1	20.5 ± 2.9	14.4 ± 2.2	29.4 ± 7.7	17 ± 1.8	[86]

^a^ EC_50_: Concentration of compounds required to achieve 50% protection of MT-4 cell cultures against HIV-1-induced cytotoxicity, as determined by the MTT method.

**Table 39:** Activity of thieno[3,2-*d*]pyrimidine derivatives **92a–k** against HIV-1 (IIIB) and HIV-2 (ROD) strains in MT-4 cells.

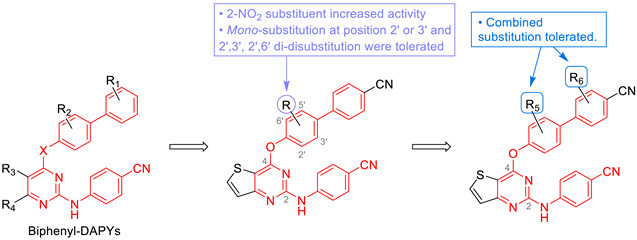						
Compound	R	R_5_	R_6_	EC_50_^a^		Ref.
				HIV-1 (IIIB) (nM)	ROD (µM)	
**92a**	2′-NO_2_	-	-	7.8 ± 3.7	>107.2	[28]
**92b**	3′-NO_2_	-	-	87.7 ± 30.6	>34.4	[28]
**92c**	2′,3′-(Me)_2_	-	-	38 ± 12.7	1.4 ± 0.3	[28]
**92d**	2′,3′-F_2_	-	-	13.5 ± 5.6	>104.3	[28]
**92e**	2′,6′-F_2_	-	-	12.3 ± 2.1	>37.8	[28]
**92f**	2′-Me-6′-Cl	-	-	34.5 ± 16.2	>70.3	[28]
**92g**	-	H	2-F	11 ± 8	-	[92]
**92h**	-	3-Me	2-F	29 ± 5	-	[92]
**92i**	-	3-Cl	3-Cl	21 ± 10	-	[92]
**92j**	-	2-Me	2-Me	14 ± 5	-	[92]
**92k**	-	2-Me	3-Me	17 ± 12	-	[92]
**NVP**	-	-	-	309.4 ± 57.7	>4.0	[28]
**ETV**	-	-	-	5.5 ± 4.1	>2.0	[28]

^a^ EC_50_: The effective concentration required to protect the cell against viral cytopathic by 50% in MT-4 cells.

**Table 40:** Activity of thieno[3,2-*d*]pyrimidine derivatives **92** against clinical HIV-1 mutant strains in MT-4 cells.

Cpd	EC_50_ ^a^ (nM)							Ref.
	L100I	K103N	Y181C	Y188L	E138K	F227L + V106A	K103N + Y181C	
**92a**	18.2 ± 3.9	5.5 ± 0.6	55.1 ± 0.6	>15,140	6.5 ± 0.5	≥40,000	>20,000	[28]
**92c**	23.2 ± 4.2	33.8 ± 4.2	57.1 ± 10.6	50.7 ± 17.0	42.3 ± 4.2	253.6 ± 8.5	152.2 ± 42.3	[28]
**92d**	52.0 ± 8.3	9.4 ± 2.1	58.2 ± 33.3	228 ± 41.6	17.0 ± 0.2	603 ± 21	>2037.1	[28]
**92e**	47.8 ± 8.3	8.7 ± 2.7	120.6 ± 18.7	>20,308	16.6 ± 2.1	≥7233.70	>78,552.4	[28]
**92f**	28.4 ± 16.2	30.4 ± 6.1	48.7 ± 14.2	107.5 ± 42	52.7 ± 6.1	405.6 ± 6.1	148.0 ± 20.3	[28]
**92g**	610 ± 270	90 ± 10	630 ± 490	-	130 ± 110	-	-	[92]
**92h**	440 ± 450	210 ± 130	710 ± 10	-	400 ± 70	-	-	[92]
**92i**	940 ± 50	>20,000	660 ± 90	-	40 ± 30	-	-	[92]
**92j**	130 ± 80	20 ± 10	30 ± 10	-	40 ± 10	-	-	[92]
**92k**	1970 ± 660	130 ± 10	>19000	-	160 ± 120	-	-	[92]
**NVP**	2102 ± 751	>10,075.0	>15,866.7	>15,866.7	210 ± 26	>15,866.7	>15,866.7	[28]
**ETV**	7.1 ± 2.8	3.2 ± 0.5	12.0 ± 1.4	20.0 ± 7.6	6.5 ± 5.8	15.2 ± 16.1	55.3 ± 9.2	[28]

^a^ EC_50_: The effective concentration required to protect the cell against viral cytopathic by 50% in MT-4 cells.

**Table 41:** Hydroxythienopyrimidine activities against RT, INST, and anti-HIV-1 cells.

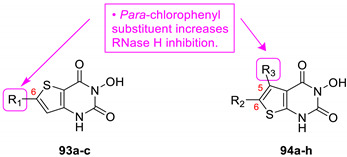								
Compounds	R_1_	R_2_	R_3_	RT IC_50_ ^a^ (μM)		INST IC_50_ ^a^ (μM)	MAGI Antiviral	
				RNase H	Pol		EC_50_ ^b^ (μM)	CC_50_ ^c^ (μM)
**93a**	Ph	-	-	0.10 ± 0.06	>10	4.5 ± 0.8	11 ± 2	28 ± 0.2
**93b**	4-F-Ph	-	-	0.20 ± 0.1	>10	17 ± 4	>20	>100
**93c**	4-Cl-Ph	-	-	0.070 ± 0.05	>10	23 ± 6	>20	>100
**94a**	-	Ph	H	0.084 ± 0.006	>10	2.2 ± 0.4	>20	>100
**94b**	-	Benzyl	H	0.043 ± 0.008	>10	5.0 ± 1	>20	>100
**94c**	-	H	Ph	0.10 ± 0.03	>10	1.3 ± 0.1	14 ± 1	>100
**94d**	-	H	4-Cl-Ph	0.040 ± 0.02	>10	2.1 ± 0.3	7.4 ± 0.3	>100
**94e**	-	H	H	0.20 ± 0.03	>10	35 ± 6	18 ± 1	54 ± 6
**94f**	-	H	Me	0.10 ± 0.02	>10	12 ± 2	>20	81 ± 6
**94g**	-	Me	Me	0.10 ± 0.03	>10	8.4 ± 1	8.9 ± 1	62 ± 1
**94h**	-	CO_2_H	Me	0.10 ± 0.02	>10	7.9 ± 1	>20	>100
**Raltegravir**	-	-	-	>10	ND ^d^	0.65	0.030 ± 0.005	ND ^d^

^a^ Concentration of compounds inhibiting the target enzyme by 50%. ^b^ Concentration of compounds inhibiting virus replication by 50%. ^c^ Concentration of compounds resulting in 50% cell death. ^d^ ND = not determined. All assay results expressed as a mean ± standard deviation from at least two independent experiments.

**Table 42:** Antiviral activity of thienopyrimidine derivatives **95a–l** against HIV-1.

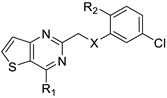				
Compound	R_1_	X	R_2_	EC_50_ ^a^ (µM)
**95a**	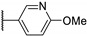	SO_2_	H	0.025
**95b**	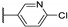	SO_2_	H	3.2
**95c**	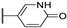	SO_2_	H	0.915
**95d**	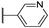	SO_2_	H	0.485
**95e**	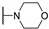	SO_2_	H	0.59
**95f**	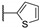	SO_2_	H	0.140
**95g**	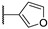	SO_2_	H	0.775
**95h**	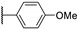	SO_2_	H	0.103
**95i**	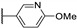	S	H	0.014
**95j**	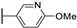	CH_2_	H	1.6
**95k**	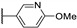	CO	H	0.20
**95l**	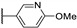	S	Cl	0.728
**Nevirapine ^b^**	-	-		0.150

^a^ EC_50_ is the concentration of compound that inhibits HIV-1 replication by 50%. The values are the geometric mean of two determinations; all individual values are within 25% of the mean. ^b^ Nevirapine was used as a positive control.

## Data Availability

Not applicable.
